# A randomized controlled trial on the effects of decision aids for choosing discharge destinations of older stroke patients

**DOI:** 10.1371/journal.pone.0272115

**Published:** 2024-01-25

**Authors:** Yoriko Aoki, Kazuhiro Nakayama, Yuki Yonekura

**Affiliations:** 1 Department of Gerontological Nursing, Faculty of Medicine, University of Toyama, Toyama, Japan; 2 Graduate School of Nursing Science, St. Luke’s International University, Tokyo, Japan; Foundation IRCCS Neurological Institute C. Besta, ITALY

## Abstract

**Background:**

In Japanese medical practice, older stroke survivors are overwhelmed with information regarding their discharge locations, creating more decision-making challenges. A randomized controlled trial evaluated the influence of decision aids (DAs) for matching older stroke patients and their families’ values concerning decisional conflict and participation in discharge destination decisions.

**Methods:**

Participants were randomly allocated to intervention and control groups. The intervention spanned two months, from admission to discharge, at which times participants were surveyed. DAs were provided to the intervention group, and brochures to the control group. The primary endpoint was decisional conflict, assessed using the Decisional Conflict Scale (DCS). The secondary endpoint decision-making participation was assessed using the Control Preference Scale (CPS) decision-making roles and a 10-point Visual Analog Scale for participation rate. An independent t-test analyzed decisional conflict scores and participation rates to examine between-group differences. The chi-square independence test evaluated roles in decision-making scores. Post hoc subgroup analyses were performed.

**Results:**

Ninety-nine participants (intervention group n = 51; control group n = 48) were included in the full analysis set, with a dropout rate of 38.4%. No significant group differences were found in decision-making conflict [t (99) = 0.69, p = 0.49, d = 0.14] and roles in decision-making scores [χ2 (5) = 3.65, p = 0.46]. However, a significant group difference was found in the participation rate [t (99) = 2.24, p = 0.03, d = 0.45]. DA tended to reduce uncertainty and promote participation rates, especially in participants living alone and unable to decide their discharge destination.

**Conclusions:**

The use of DA with older stroke patients did not significantly decrease decisional conflicts. In addition, the participation rate in decision-making increased, but their active role did not. Further studies should be conducted to understand the methods of offering DA, their ideal durations, and identify their beneficiaries.

## Introduction

Medical care for strokes has advanced, and its mortality rates have drastically declined. However, age-related morbidity and recurrence rates of strokes remain high, with strokes being the second most common condition after dementia to lead to patients requiring long-term nursing care [1]. As a result, the roles of care personnel and need for recovery rehabilitation to enable patients to live independently have intensified. However, individuals who have suffered a stroke experience such drastic changes in their lives that an internal conflict arises between their past self (that is hard to let go of) and their shattered self-image [2]. Therefore, numerous factors must be considered when selecting their discharge destination, including the role of their families, cognitive aspects, individual patient care behaviors and activities, health status, age, and income [3]. Patients then face a dilemma about their post-discharge destination: to continue living at home or to receive care at a different location. In Japan, older people are often cared for by their families. Therefore, post-discharge destination decisions are often finalized between the family and healthcare professionals without any input from the older patients [4]. The reasons include difficulties in communicating with older patients due to the severity of their condition and their families’ mindset that the older patient’s participation in decision-making is unnecessary [5]. As a result, hospitals face the challenge of coordinating among older patients, their families, and healthcare professionals to adjust the “divergences in intentions as to discharge destination” [6]. However, there have been no established methods of aiding decision-making and no assessment criteria for decisions until now in Japan. Therefore, older patients and their families are at risk of being stricken with anxiety and remorse about the decisions made [7, 8]. The practice of shared decision-making (SDM) [9], in which patients and physicians are involved in making medical decisions together, is gradually being adopted at clinical sites. Moreover, an improved version of SDM called the international professional SDM (IP-SDM) model [10], has been developed; it includes families and multidisciplinary professionals in the decision-making process. This multi-professional approach has been reported as helpful when making post-discharge destination decisions [11]. One method of aiding decision-making that the IP-SDM model promotes is the use of decision aids (DAs). Numerous DAs have been developed overseas and are being adopted as decision-making tools [12]. Unlike conventional informative materials, DAs compare the advantages and disadvantages of various choices and encourage choosing those that match a person’s values [13]. Some effects that have been confirmed so far and reported in all populations include increased knowledge, decreased ambiguity of internal conflicts and values, and increased participation in decision-making [14]. They have also proven to be equally effective for older people [15]. However, in the case of older people, due to reasons such as frailty and dementia, it is not easy to develop DA [16], and progress has been limited. In Japan, patients are given informative brochures upon hospital discharge. The massive amounts of information in these booklets can be overwhelming, creating further challenges in decision-making [17]. Unfortunately, no recommended DA can be used by patients, families, and professionals together. Thus, the likely effectiveness of such DAs has not been established.

Therefore, this study aimed to use a randomized controlled trial (RCT) to evaluate the influence of the use of DAs that match the values of older stroke patients and their families on the decisional conflict and participation in discharge destination decisions. We hypothesized that the group that is provided with a DA in selecting a discharge location will have significantly reduced decision-making conflict and increased decision-making participation compared to the non-intervention group.

## Materials and methods

This study was conducted according to a predetermined protocol [18]. Consent to participate in the study was obtained through each participant’s signature on a consent form after receiving an explanation regarding the study, both verbally and by written documents. Furthermore, the research was conducted with the approval of the Ethics Review Committee of St. Luke’s International University (18-A010, recognized on July 3, 2018), Toyama University (C 30–60, recognized on August 3, 2018), and the Children’s Support Centre of Toyama Prefecture Rehabilitation Hospital (No. 51, recognized on August 15, 2018). The study was registered in May 2018. Patient recruitment began in October of the same year, and data collection continued until May 2020, when COVID-19 regulations prohibited entry into the hospital. The study was temporarily halted for follow-up, and further recruitment of participants was terminated in March 2021 as resuming the data collection was not feasible.

### Study design

This study performed a two-arm parallel RCT, based on the Ottawa decision support framework [19]. This was a single-center, single-blinded test with participants allocated to the intervention group and the control group at a 1:1 ratio. The entire trial complied with CONSORT (Consolidated Standards of Reporting Trials) guidelines [20, 21], met the requirements of the CONSORT checklist (S1 Checklist), and thus conformed to the definition of a randomized test. This trial was registered with the University Hospital Medical Information Network (UMIN Registration No.: UMIN000032623, May 17, 2018), certified as a test registration institution by the World Health Organization. The authors confirm that all ongoing and related trials for this intervention are registered.

### Setting

Toyama Prefectural Rehabilitation Hospital and Support Center for Children with Disabilities was the sole institution that participated in the research. The facility has 100 beds, 50 each in the third and fourth floors. The personnel who usually provide discharge assistance are physicians, nurses, physical therapists, occupational therapists, and medical social workers. While dividing roles among themselves, these multidisciplinary professionals ask older stroke patients and their families about the discharge destinations of their choice. Based on their wishes, they narrow options to two or three potential facilities and social welfare services and then propose them to the patients. The staff holds numerous meetings and offers explanations orally as needed while handing out the brochures issued by the facility and municipalities. This discharge assistance method leaves the decision to multidisciplinary professionals and focuses on providing information about limited choices. Moreover, the materials offered contain vast amounts of information, such as an overview of the facilities and social welfare services. They lack content that would aid decision-making, such as the types of choices available and information on their advantages and disadvantages.

### Participants

The research participants were as follows: (1) older persons aged 65 and older, (2) those who had suffered a stroke (cerebral infarction, cerebral hemorrhage, or subarachnoid hemorrhage), and (3) those admitted to rehabilitation wards during their convalescence and who had to decide their location of care after discharge. However, we excluded individuals with severe dementia or aphasia and those facing difficulty making decisions because of an altered state of consciousness.

### Enrolment and allocation

Based on the prescribed facility criteria and preliminary survey [22], we found that the third and fourth floors were similar and concluded the baseline conditions to be the same for both in terms of patient gender, age, severity of illness, and the ratio of the number of stroke patients. Approximately two weeks after admission to the rehabilitation hospital, when the patients had familiarized themselves with their hospital environment, those who met the eligibility criteria were introduced to us by the head nurse. The principal investigator described the outline of the study orally to the patients, using an explanatory document. The participants were enrolled in the study after they provided written informed consent. While following the allocation table, the principal investigator randomly allocated the participants to the intervention or the control groups according to the hospital room where the initial meeting with the research participants had occurred. The principal investigator created a table by integrating (a) a random number table that Research Assistant A had created using a computer at a 1:1 ratio and (b) an allocation table of patients according to their condition’s severity designed by the ward’s head nurse. The severity of illness was determined by the lowest total score of a daily living function assessment [23, S1 File] and the Functional Independence Measure (FIM) [24]. According to the facility criteria prescribed by the government, the severely ill are those who have a daily living function assessment of 10 points or more, or a total FIM score of 55 points or less. Until the allocation to the groups was completed, the order of allocation was concealed from Research Assistant A, the floor’s head nurse, the patients, their families, and multidisciplinary professionals (as part of the “allocation concealment mechanism”).

### The flow of selecting participants

**Fig 1:**
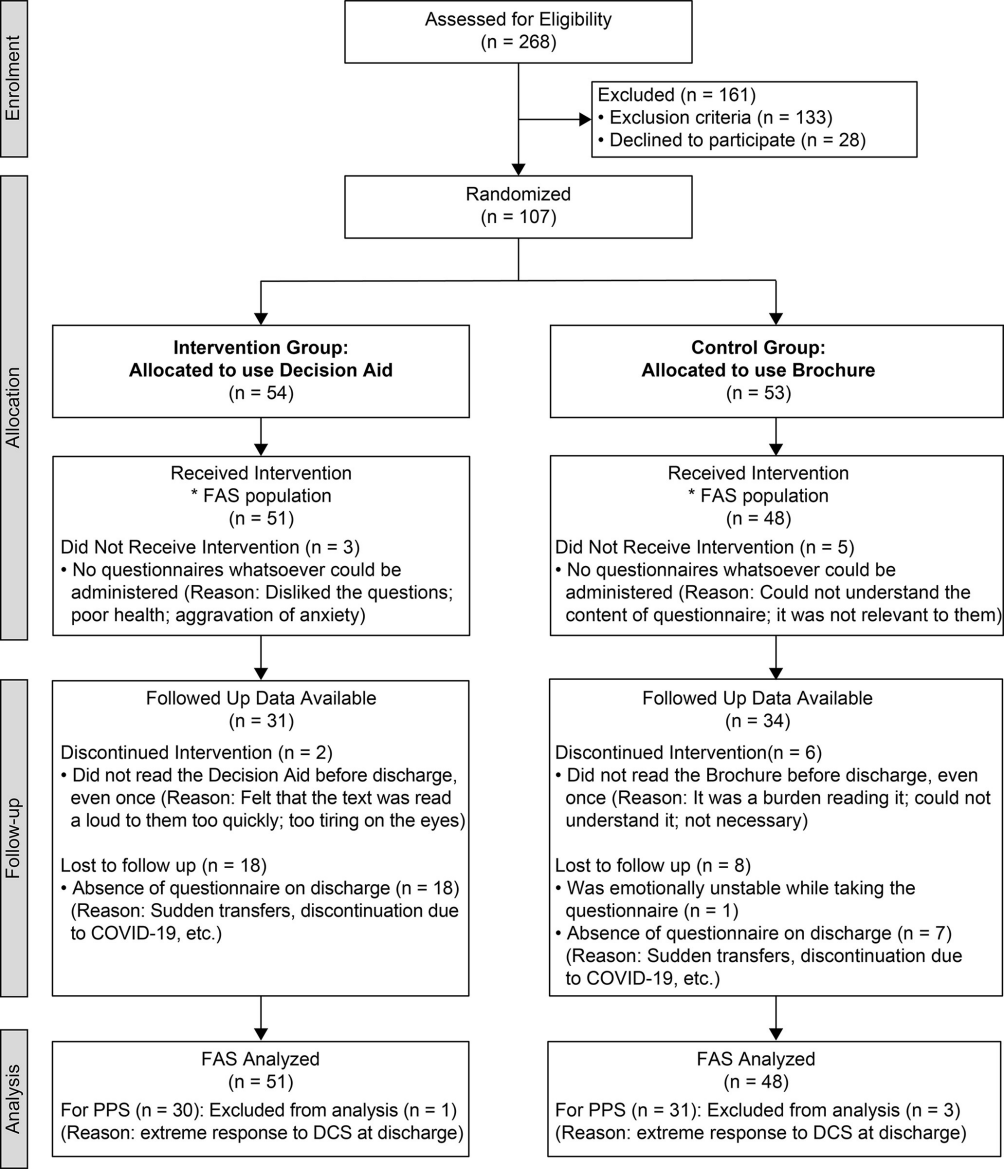
Flow diagram of the CONSORT study. The figure describes the stages of the CONSORT study, beginning with the enrolment of the participants, followed by their allocation into intervention and control groups, their follow-up, and finally the analysis of the two groups.

Between October 2018 and May 2020, 268 patients aged 65 years and older who had suffered a stroke were selected as potential research collaborators. Among them, we excluded 133 patients who did not meet the eligibility criteria (those with severe dementia, aphasia, or altered state of consciousness, as these conditions would cause difficulty in decision-making). Exclusion criteria were based on higher scores on the five cognitive items of the FIM measured at admission (all items were rated on a 7-point scale ranging 1–7, with higher scores indicating greater independence) and the overall clinical judgment of the head nurse on the floor. The breakdown is as follows: comprehension 7–5 (able to understand questions of daily living), expression 7–4 (able to speak in short sentences about basic daily living needs), social interaction 7–5 (no difficulty in participating in training), problem solving 7–5 (able to make requests regarding daily living), and memory 7–5 (able to keep track of the daily schedule, but sometimes forgets). No strict minimum score was set because some patients become able to communicate their intentions after 2 weeks of hospitalization (i.e., when the questionnaire survey is administered), having become familiar with the hospital environment; patients with the highest possible score and ability to communicate their intentions were selected. We invited 135 individuals who had met the eligibility criteria to take part in the trial. After excluding those who had declined to take part (n = 28), we randomly allocated 107 individuals to the intervention or the control groups. Furthermore, eight individuals were excluded with whom, in the course of follow-up, no questionnaire survey could be carried out. Finally, 99 people, comprising 51 in the intervention group and 48 in the control group, were included in the full analysis set (FAS), with a dropout rate of 38.2% (Fig 1).

Although we planned to enroll 122 patients, we were not allowed to enter the hospital because of the COVID-19 pandemic; hence, we halted the process temporarily. Given that the situation remained unchanged, even after a year, we decided to carry out an analysis using the number of patients we had obtained up to that point.

### Intervention method

Following Coulter’s [25] systematic development 6 process, a DA was developed based on the 47-item international patient DA standards instrument [26]. DAs consisting of 12 A4 pages were developed, listing the following six values that were common to older stroke patients and their families: (1) living standards, (2) services and costs, (3) emergencies, (4) family support, (5) environment, and (6) home repair and renovation [27, S2 and S3 Files].

Based on the prescribed facility criteria and preliminary survey [22], the mean length of hospital stay was 86.1 ± 44.3 days. Therefore, the intervention period for both groups was approximately 2 months from admission to discharge. With the help of Research Assistants B (this included four research assistants who had similar roles but worked separately, as required for the study), we conducted a questionnaire survey twice, once on admission to rehabilitation and once at discharge. The research participants and their families, the floor’s head nurse, multidisciplinary professionals, and Research Assistants B were blind to the intervention.

A month after admission to rehabilitation, we enquired with the intervention group regarding the usage of the DAs and whether they had received them. Approximately two weeks after admission, the principal investigator offered DAs to the participants in a private room. The principal investigator explained the purpose of the DA, its content, method of use, and points to note. The principal investigator ensured that the participants understood the advantages and disadvantages of the two possible post-discharge destinations, namely “the same place as before admission” and “a place different from before admission.” The investigator explained to them that the purpose of the DA was to assist them in making decisions that suited their circumstances and values. The principal investigator also explained that the content of the DA consisted of (1) information to help with devising a discharge plan, (2) information on the types and characteristics of services available, (3) information about the advantages and disadvantages of the discharge destination, (4) help with judging important values, and (5) help with organizing hospital discharge after preparations for discharge have been completed. The principal investigator explained that the patients could read the DA whenever they wished to prepare themselves for discharge and use it with their families and multidisciplinary professionals if needed. The participants were reminded not to share and show the booklets to other patients within the floor or their families by explaining to them that the effectiveness of DAs has not yet been established, and so two types of booklets have been handed out to all the patients to investigate their effectiveness. Approximately one month after admission (about two weeks after the DA was provided), after discussing the future course of action with a physician, a 15-minute interview was conducted privately to understand how the patients were using the DAs. We asked the patients if they had read the DA, used it with their family or multidisciplinary professionals, and had any questions about the content and method of using the DA. After a month of admission, we enquired with the control group regarding the usage of the brochures (given in place of DAs) and if they had received them. The brochure’s content, describing the type and characteristics of the services available, was similar to that of the DA. The participants were explained that the brochure had been provided for their reference while deciding their discharge destinations and that it contained the same methods of usage and points of special note as those provided to the intervention group.

We held two meetings with the multidisciplinary professionals to explain the purpose, significance, and method of research. We explained that we could not reveal the contents of the DA or the brochure, or the allocation of patients between the two groups. We also informed them that they may respond to the queries of the older stroke patients and their families but should avoid providing instructions regarding the content of DA or about utilizing the tool. Furthermore, we asked licensed nurses and fourth-grade nursing university students who had completed their practical training to serve as Research Assistants B in conducting the questionnaire survey together. Furthermore, we trained them using a manual developed by the authors to ensure that they could provide standard and appropriate answers to anticipated questions from the research participants (S4 File). We explained to them that we cannot reveal the allocation of the patients and that they are not allowed to look at the content of the DA or the brochures throughout the study duration.

### Evaluation items

The primary and secondary endpoints were evaluated, on admission and at discharge, together with Research Assistants B, via a questionnaire survey. The primary endpoint pertained to decisional conflict and was evaluated using the 16-item Japanese-language edition of the decisional conflict scale (DCS) [28]. The DCS was developed by O’Connor [29], and it is a highly reliable scale for identifying the intervention effects of DAs. The test-retest reliability coefficient was 0.81, and the internal consistency coefficients ranged from 0.78 to 0.92. The Japanese-edition DCS also shows high internal consistency (Cronbach’s α: 0.84–0.96) [28]. DCS comprises five items, namely sufficient explanation of information, clarification of values, support, uncertainty, and effective decision-making. Each item is evaluated using a 5-point Likert scale. All the DCS items are totaled, divided by 16, and multiplied by 25 to arrive at the total score. The total score is converted into a score ranging from 0 to 100 points, with a high score indicating a high decisional conflict level. A score below 25 points indicates implementation of decision-making, and a score of 37.5 points or higher indicates a delay in decision-making and a feeling of uncertainty about its implementation [30].

The secondary endpoint pertained to participation in decision-making and was evaluated using two scales: the control preference scale (CPS) for roles in decision-making and the Visual Analog Scale for participation rates. The CPS was developed by Strull et al. [31] and modified by Degner et al. [32]. Its reliability has been confirmed (Coombs’ criterion of 50%). The reliability of the Japanese edition of CPS has also been confirmed. The test-retest reliability of the kappa coefficient was 0.61; the weighted kappa coefficient was 0.61; and Kendall’s tau coefficient was 0.61 [33]. The role in decision-making desired by the participant is evaluated using the five written answers. Answers to Choices 1 and 2 are classified as “Active roles” (decision-making by the self), Choice 3 is classified as “Shared roles” (SDM),” and Choices 4 and 5 are classified as “Passive roles” (decision-making by others). The percentages of participation rates were also calculated using a 10-point Visual Analog Scale, measured from 0–100%.

We also asked for several details regarding the participants’ attributes, on admission: sex, age, disease name, family structure, the desired and ultimate discharge destination, education, work history, duration of hospitalization, the status of readiness for decision-making [34], and the person(s) with whom a decision had been made.

### Calculation of sample size

The sample size was calculated based on the effect size of 0.3–0.4 from past studies whose primary endpoint was the DCS in the systematic review of a patient’s DAs [14]. Effect sizes of 0.4 to 0.8 on the DCS scale are meaningful in clinical practice, allowing for differences between groups with smooth and slow decision-making to be identified [30]. Therefore, we assumed that 61 individuals were needed per group by considering a power of 0.80, an effect size of 0.5, a level of significance of two-sided α of 0.05, and losses to follow-up of 20%.

### Method of analysis

After checking the data that were input by two Research Assistants B, the primary investigator, who was not blind to the allocation process, handled the data. We analyzed the FAS, which excluded only cases that had no post-randomization (S5 File). All participants’ characteristics at baseline underwent descriptive statistical testing, and a *t*-test and χ^2^-test were used to verify differences between intervention and control groups. The decisional conflict (the primary endpoint) was subjected to an independent t-test to compare the inter-group number of changes in the DCS subscales between the time of admission and the time of discharge. Additionally, a paired t-test was performed to assess the mean at the time of hospital admission (baseline) and at the time of discharge. A multiple regression analysis was also carried out to adjust the baseline values. Regarding participation in decision-making (the secondary endpoint), a chi-squared test of independence was conducted to examine the differences in the inter-group ratios of the roles in decision-making (CPS), and another chi-square test was conducted to examine the differences in the ratio between the time of admission and the time of discharge. An independent t-test was conducted to make inter-group comparisons between participation rates, and a paired t-test was conducted to compare the temporal differences in the time of admission and discharge. An ad-hoc subgroup analysis [18] was also conducted for those experiencing intense decisional conflict (DCS of 37.5 points or higher on admission), those living alone, adults aged 75 and older (higher rates of receiving care [35]), those undecided about their discharge destination concerning their readiness for decision-making at the time of admission, and those experiencing extended hospitalization (average duration of hospitalization: 78 days or more). Subgroup analyses were conducted using either parametric or non-parametric methods (the Mann–Whitney U test was applied for decisional conflict scores and participation rates, the Fisher’s exact test for roles in decision-making) depending on the data distribution. Multiple imputations were made in the case of missing values. SPSS Statistics for Windows, version 28 (IBM Corp., Armonk, N.Y., USA), was used for statistical analysis, and the level of significance was made two-sided, 5% or less.　

## Results

### Characteristics of the participants

Table 1 summarizes the characteristics of the participants at baseline. The participants were hospitalized for an average duration of 73.2 days (SD = 35.6) for the intervention group and 87.4 days (SD = 43.4) for the control group. The average age of the participants was 75.0 years (SD = 6.4) in the intervention group and 75.5 years (SD = 6.6) in the control group. In terms of the status of readiness for decision-making, in the intervention group, 66.7% had already decided on their discharge destination and 45.8% had done so in the control group. The discharge destination was the same place as before admission in 65.7% of the participants and a different place in 34.3% of the participants. Regarding whom the participants wanted to decide their discharge destination, the largest number of the participants wanted to do so “With their family,” followed by “With family and healthcare professionals.”

**Table 1:** Characteristics of participants at baseline.

Survey item	Intervention Group (n = 51)		Control Group (n = 48)		*p-value*
Duration of hospitalization	73.2	(35.6)	87.4	(43.4)	*0*.*08*
Age	75.0	(6.4)	75.5	(6.6)	*0*.*69*
Sex					
Male	32	(62.7)	32	(66.7)	*0*.*83*
Female	19	(37.3)	16	(33.3)	
Disease name					
Stroke	36	(70.6)	34	(70.8)	*0*.*99*
Cerebral hemorrhage	12	(23.5)	11	(22.9)	
Subarachnoid hemorrhage	3	(5.9)	3	(6.3)	
Family makeup					
Living alone	10	(19.6)	12	(25.0)	*1*.*00*^*a)*^
Living with one more person	23	(45.1)	18	(37.5)	
Living with two other people	13	(25.5)	11	(22.9)	
Living with three other people	3	(5.9)	2	(4.2)	
Living with four or more people	2	(3.9)	5	(10.4)	
Work history					
Corporate employee	29	(56.9)	26	(54.2)	*0*.*84*^*b)*^
Self-employed	10	(19.6)	8	(16.7)	
Public employee	1	(2.0)	1	(2.1)	
Healthcare	2	(3.9)	1	(2.1)	
Welfare	0	(0.0)	1	(2.1)	
Part-time worker	2	(3.9)	2	(4.2)	
Housewife	3	(5.9)	4	(8.3)	
Agriculture	3	(5.9)	4	(8.3)	
Others	1	(2.0)	1	(2.1)	
Education					
Graduated from elementary school in the old educational system (1886–1941)	2	(3.9)	5	(10.4)	*1*.*00*^*c)*^
Graduated from a girls’ school in the old educational system (1886–1941)	0	(0.0)	0	(0.0)	
Graduated from middle school	9	(17.6)	5	(10.4)	
Graduated from high school	28	(54.9)	25	(52.1)	
Graduated from a vocational college	3	(5.9)	6	(12.5)	
Graduated from junior college	1	(2.0)	0	(0.0)	
Graduated from university	8	(15.7)	7	(14.6)	
Graduated from graduate school	0	(0.0)	0	(0.0)	
Status of readiness for decision-making					
1. Cannot even begin to think about a discharge destination	10	(19.6)	14	(29.2)	*p<0*.*05*^*d)*^
2. Not even considering discharge destinations other than the one desired	5	(9.8)	5	(10.4)	
3. Also considering discharge destinations other than the one desired	0	(0.0)	4	(8.3)	
4. On the verge of deciding where to go to after discharge	2	(3.9)	3	(6.3)	
5. Have already decided where to go to after discharge	34	(66.7)	22	(45.8)	

Mean (standard deviation), No. of people (%).

Multiple imputations were made in the case of missing values.

p-value: Independent t-test for the duration of hospitalization and age on admission; chi-squared test for sex, disease name, family makeup, work history, education, and the status of readiness for decision-making.

a) Family makeup: "Living alone" and "Other than living alone," which includes living with one more person, living with two other people, living with three other people, and living with four or more people.

b) Work history: "Corporate employee" and "Other than a corporate employee". indicates self-employed, public employee, healthcare worker, welfare staff, housewife, agriculturist, and others.

c) Education: Regarding "Graduated from schools below high school" and "Graduated from schools above high school," "Graduated from schools below high school" indicates having graduated from elementary school in the old educational system, graduation from a girls’ school in the old educational system, and graduation from middle school. "Graduated from schools higher than high school" indicates having graduated from high school, vocational college, junior college, university, and graduate school.

d) Status of readiness for decision-making: "Have already decided where to go to after discharge" and "Have not decided where to go to after discharge," which includes "Cannot even begin to think about a discharge destination," "Not even considering discharge destinations other than the one desired," "Also considering discharge destinations other than the one desired," and "On the verge of deciding where to go to after discharge."

We found that the intervention group contained significantly more participants who had already decided their discharge destination than the control group (*p* < 0.05) (Table 1). It was also found that significantly more participants chose “the same place as before admission” as their discharge destination (p < 0.01) (Table 2).

**Table 2:** Discharge decision characteristics.

Survey item	Intervention Group (n = 51)				*p-value (before/after difference)*	Control Group (n = 48)				*p-value (before/after difference)*	*p-value (inter-group difference)*
	On admission		At discharge			On admission		At discharge			
Discharge destination					* *					* *	
Same place as before admission	48	(94.1)	36	(70.6)	*0*.*20*	42	(87.5)	29	(60.4)	*p<0*.*01*	*p<0*.*01*
Different place from before admission	3	(5.9)	15	(29.4)		6	(12.5)	19	(39.6)		
With whom the subjects want to decide their discharge destination											
By themselves	3	(5.9)	3	(5.9)	*P<0*.*05*^*a)*^	2	(4.2)	1	(2.1)	*0*.*89*^*a)*^	*0*.*09*^*a)*^
With family	34	(66.6)	21	(41.2)		33	(68.7)	34	(70.8)		
With healthcare professionals	0	(0.0)	9	(17.6)		1	(2.1)	4	(8.3)		
With family and healthcare professionals	14	(27.5)	18	(35.3)		11	(22.9)	9	(18.8)		
Want to leave it to XX	0	(0.0)	0	(0.0)	* *	1	(2.1)	0	(0.0)	* *	

Number of people (%). Multiple imputations were made in the case of missing values.

p-value: In a test of the ratio’s before/after differences, a chi-square test was performed for "discharge destination" and "With whom the subjects wanted to decide their discharge destination;" and a chi-squared test of independence was performed for testing inter-group differences.

a) "With whom the subjects want to decide their discharge destination" was studied with three items: "By themselves," "With family," and "With family and healthcare professionals."

### Primary endpoint

In the decisional conflict (measured by the DCS), both the intervention and control groups had intense internal conflict over “Support,” “Sufficient explanation of information,” and “Clarification of values.” The decisional conflict continued even at the time of discharge. On the contrary, the level of decisional conflict over “Effective decision-making” was the lowest measurement found during admission and discharge. No significant inter-group differences were seen in terms of the extent of change in DCS scores between admission and discharge [t (99) = 0.69, p = 0.49, d = 0.14] (Table 3).

**Table 3:** Comparison of changes in decision-making conflict.

Survey item		Intervention Group (n = 51)			Control Group (n = 48)			Amount of Change					
		mean	SD	*p*-value	mean	SD	*p*-value	mean	SD	*t*-value	95%	*p*-value	*d*
											CI		
*DCS total score	On admission	47.15	18.57	*p<0*.*01*	51.95	21.49	*p<0*.*01*	–13.70	14.01	0.69	–5.43, 11.19	*0*.*49*	*0*.*14*
	At discharge	33.45	17.52		35.37	19.63		–16.58	25.48				
Sufficient explanation of information	On admission	53.43	32.24	*p<0*.*01*	56.08	31.59	*p<0*.*01*	–13.25	29.20	0.35	–10.59, 15.04	*0*.*73*	*0*.*07*
	At discharge	40.18	30.72		40.60	26.58		–15.48	34.92				
Clarification of expectations/value	On admission	45.75	24.74	*0*.*30*	52.43	25.32	*p<0*.*05*	–4.50	30.99	1.29	–4.72, 22.25	*0*.*20*	*0*.*26*
	At discharge	41.25	23.27		39.16	24.70		–13.27	36.52				
Support	On admission	59.15	22.25	*p<0*.*01*	59.03	24.12	*p<0*.*01*	–18.01	19.57	0.37	–9.02, 13.07	*0*.*72*	*0*.*07*
	At discharge	41.14	22.89		39.00	24.15		–20.03	33.39				
Uncertainty	On admission	43.14	28.47	*p<0*.*01*	53.65	28.91	*p<0*.*01*	–17.74	21.00	0.40	–8.59, 12.91	*0*.*69*	*0*.*08*
	At discharge	25.40	26.23		33.75	26.60		–19.90	31.39				
Effective decision-making	On admission	37.50	21.90	*p<0*.*01*	41.93	25.82	*p<0*.*01*	–14.67	18.67	0.03	–8.68, 8.95	*0*.*98*	*0*.*01*
	At discharge	22.83	19.80		27.12	21.74		–14.81	25.23				

To examine the intervention effects of DCS and participation rates, an independent t-test was performed for the amount of change (mean at discharge—mean on admission)

A paired t-test was performed for the mean on hospital admission (baseline) and the mean at discharge.

Mean (mean), SD (standard deviation). Multiple imputations were made in the case of missing values.

Cohen’s d shows the effect size, and the yardstick for indices was effect size large: d = 0.80, effect size medium: d = 0.50, and effect size small: d = 0.20.

*DCS: Decision conflict scale

Regarding “uncertainty,” in particular, the number of participants who were undecided in terms of the status of readiness for decision-making showed a significantly high score *(p* < 0.05) (Table 4).

**Table 4:** Comparison between the number of changes in decisional conflict and multiple regression analysis results.

Survey item	*DCS’ amount of change (n = 99)											
	Total score		Sufficient explanation of information		Clarification of value		Support		Uncertainty		Effective decision-making	
	^§^B	*p*-value	B	*p*-value	B	*p-*value	B	*p-*value	B	*p-*value	B	*p-*value
Duration of hospitalization	–0.01	*0*.*83*	–0.03	*0*.*64*	–0.04	*0*.*54*	–0.04	*0*.*50*	0.03	*0*.*58*	0.04	*0*.*46*
Status of readiness for decision–making	4.82	*0*.*23*	6.40	*0*.*26*	5.48	*0*.*31*	0.29	*0*.*95*	13.21	*p<0*.*05*	1.13	*0*.*80*
Presence/absence of intervention	–0.69	*0*.*84*	–1.47	*0*.*79*	–2.87	*0*.*57*	–1.56	*0*.*74*	1.25	*0*.*78*	1.66	*0*.*66*
Adjusted coefficient of determination R^2^	0.32		0.34		0.49		0.31		0.32		0.33	

A multiple regression analysis was conducted, using the subitem of DCS’ amount of change (at discharge- on admission) as the dependent variable, controlling it with the DCS’ baseline values, and using the duration of hospitalization, the status of readiness for decision-making, and presence/absence of intervention as the independent variables.

Consecutive numbers were used for DCS and duration of hospitalization, and dummy variables were used for qualitative variables. Analysis was then performed, using the status of readiness for decision-making (1: Have already decided, 2. Not yet decided), and presence/absence of intervention (1. Yes, 2. No).

§B = Non-standard partial regression coefficient. Multiple imputations were made in the case of missing values.

*DCS: Decisional conflict scale

### Secondary endpoint

In terms of participation in decision-making, the roles in decision-making as measured by the CPS, both the intervention and control groups gave the highest scores for “Active roles.” However, no significant differences were seen between the groups [χ^2^ (5) = 3.65, p = 0.46] (Table 5). On the other hand, there was a significant group difference in participation rates [t (99) = 2.24, p = 0.03, d = 0.45] (Table 6).

**Table 5:** Comparison of participation in decision-making.

Survey item	Intervention Group (*n* = 51)				*p*-value (before/after difference)	Control Group (*n* = 48)				*p*-value (before/after difference)	*p*-value (inter-group difference)
	On admission (*n* = 51)		At discharge (*n* = 51)			On admission (*n* = 48)		At discharge (*n* = 48)			
	n	%	n	%		n	%	n	%		
*CPS											
Active role	28	(54.9)	27	(52.9)	*0*.*64*	27	(56.3)	30	(62.5)	*0*.*43*	*0*.*46*
Cooperative role	19	(37.3)	16	(31.4)		11	(22.9)	9	(18.8)		
Passive role	4	(7.8)	8	(15.7)		10	(20.8)	9	(18.8)		

n stands for no. of people; % shows percentages. Multiple imputations were made in the case of missing values.

p-value: A chi-square test was performed to examine the before/after differences in the ratio of CPS’ roles in decision-making, and a chi-squared test of independence was performed for testing inter-group differences.

*CPS: Control preference scale

**Table 6:** Differences in the mean participation rates between intervention and control groups.

	Intervention group (*n = 51*)		Control group (*n = 48*)		*t-value*	95%CI	*p-value*	d*
	mean	SD	mean	SD				
Participation rate	5.19	41.30	-15.08	48.79	2.24	2.28, 38.26	*P<0*.*05*	*0*.*45*

An independent t-test was conducted to evaluate the results of the participation rate intervention, focusing on the amount of change (mean value at discharge—mean value on admission). The mean and SD are presented. Multiple imputations were made in the case of missing values. *d indicates effect size, and index criteria were as follows: large effect size: d = .80, medium effect size: d = .50, and small effect size: d = .20.

### Subgroup analysis

In terms of the effect size of the amount of change in DCS scores, a moderately significant tendency was observed with “uncertainty” [Z (22) = –1.94, p = 0.05, *r* = 0.41] and “effective decision-making” [Z (22) = –2.24, p = 0.03, *r* = 0.48] in people who were living alone and with “clarification of values” [t (49) = 1.90, p = 0.06, d = 0.55] in older adults aged 75 and older.

Concerning the effect size of the amount of change in the participation rate, a moderately significant tendency was seen among participants living alone [Z (22) = -2.08, p = 0.04, *r* = 0.44].

## Discussion

This study examined the use of DAs based on the values held by older stroke patients and their families and used an RCT to evaluate their influence on discharge destination decisions, decisional conflict and participation. Regarding decisional conflict (DCS), no significant reductions in scores were seen that were attributable to the use of DAs. A tendency to be satisfied with decision-making was observed despite high decisional conflict states in sufficient explanation of information, clarification of values, and support persisting at discharge, although it was not statistically significant. It has been reported that the place of convalescence desired may vary according to the participant’s condition, period, and what he/she wishes to prioritize [36]. In our study, the share of older stroke patients returning home after discharge was high—approximately 65%—which was roughly 10% higher than the share of older cancer patients [37, 38].

Moreover, older stroke patients believed, prior to hospital admission, that they would return home (which is the same place as before admission), and most felt that was the only choice available to them. Schkade and Kahneman [39] showed the tendency to use only a part of the information that may be available and to underestimate information to which they do not direct their attention while making their decisions. Thus, older stroke patients who were already satisfied with being discharged to their homes may have become confused and unable to cope with the excessive information and choices they were offered. On hospital admission, the participants’ DCS scores showed high decisional conflict states in all sub-items and significantly impacted the amount of change in scores from the time of discharge. This showed that the discharge destination decision caused older stroke patients’ intense decisional conflict. This is reported to cause strong remorse [40] and gaps/discrepancies between the patient and their family and healthcare professionals [41]. Japan followed other countries and, in 2014, specified a DCS score of over 40 points as a condition for individuals to receive Cancer Patient Management Funding II. Assessing DCS beginning with hospital admission helps select patients who should receive nursing interventions and evaluate such nursing interventions. In this study, subgroup analysis suggested that individuals who could not make decisions after hospital admission experienced intense decisional conflict and uncertainty and that DAs reduced decisional conflict caused by uncertainty, especially in people living alone. Researchers have pointed out the psychological need on the part of patients who have developed cerebrovascular disorder, a condition from which recovery is difficult to predict, and their families, to grasp the prospects of their home convalescent care [42]. In addition to this uncertainty of visualizing the future, of not being able to see the light at the end of the tunnel, it was believed that uncertainty would increase for those living alone because of the shortage of support and assistance.

In our study, although “clarification of values” indicated the smallest amount of change from admission to discharge in comparison to the other four DCS scales, it tended to increase decisional conflict in older adults aged over 75. This finding was similar to that of Stacey et al. [14] who reported little evidence that people made choices with DA that matched their values based on information (RR: 2.06, 95% CI: 1.46–2.91). Concerning the place of convalescence for older adults, their final abode must also be considered and the grounds for determining the best place for older adults have not yet been clarified [43, 44]. This result shows that diverse values exist when deciding the place of convalescence for older adults [16, 45, 46]. In addition, older adults make decisions relying on their past experiences and predictions, making them liable to biases [47]. Older adult patients agree to return home when it is suggested, and professionals may not feel the need to try and describe other potential locations to the patients. Normal decision-making involves a process of value clarification by comparing the advantages and disadvantages of information about alternatives [48]. However, this finding suggests a risk that the advantages and, especially disadvantages, cannot be compared—which also happens to be part of the decision-making process—and that values are less liable to be clarified. Dugas et al. [49] stated that involving the immediate parties in the development process helps to avoid stigma and to clarify society’s essential problems. Therefore, the DA used in our study was developed based on the values of older stroke patients and their families who had to choose where to live after discharge. However, a factor that contributed to the lack of effect of DA was that all the values extracted were important, suggesting it was difficult to differentiate them. It was also expected that the need for a DA to present other options was low since the majority of the subjects’ initial decision for the discharge location was the same as the actual discharge location. The average life expectancy for independent living in Japan is 72.68 years for men and 75.38 years for women (2019) [35]. In addition, the average age of institutionalized residents is over 80 years old [50], although this varies for each type of facility. Therefore, it is necessary to identify the optimal target population for future use of DA.

Next, in terms of participation in decision-making, no significant increases were seen in CPS scores after using DA. However, the use of DA tended to promote participation rates. Subgroup analysis also revealed similar results for participants who lived alone. Our study’s percentage of CPS playing an “active role” was about 30% lower than that seen in past research of other countries [51]. Instead, the percentages were characteristically high in terms of “cooperative role” and “passive role,” that is, working together with other people or leaving the decision to others. However, Almborg et al. [52] report that almost none of the patients who take part in discharge planning believe that they were participating in planning for their treatment and care needs, services, rehabilitation, or goal-setting. As a result, the roles of decision-making, as evaluated by CPS, were based solely on self-reporting by older stroke patients. Hence, we feel that they have not been able to appropriately grasp whether or not they had actually participated in decision-making. As cultural characteristics of decision-making among the Japanese, Kawai et al. [53] state that people tend to emphasize harmony, deliberately refrain from stating their opinions, leave decision-making entirely to others, and provide tacit consent. However, the fact that older stroke patients had wished to decide their discharge destination, and had acknowledged that they had taken part in them, was a new insight we gained. In our study, those who had made decisions with someone else, such as family and healthcare professionals, accounted for approximately 80% and almost no one made decisions on his/her own. As seen, even if the decisions were about older adults and they had to decide where to discharge themselves, the fact that they had decided together with family and healthcare professionals may have led to their high level of awareness of participating in the process. It has been shown that the ability to take part in decision-making (as evaluated by CPS) is influenced most strongly by a shortage of knowledge of the choices available, the patients’ preferences, and a lack of balance in power relationships [54]. Older stroke patients, expecting to return home after discharge, may have hesitated to make a decision, out of a sense of guilt and awareness of having been afflicted by a stroke and that they would therefore be highly dependent on someone else. Therefore, the results suggest that DAs may be useful in promoting participation in decision-making and may be more effective, especially for those who live alone and tend to lack support.

Based on the above, it is desirable to use DA for single patients, and using it with family members and specialists can encourage their participation in decision-making. It was also thought that if the specialist carefully explained the advantages and disadvantages, the older person’s sense of value would be clarified and their anxiety about future prospects would be reduced.

### Limitations

The DA utilized in this study was the first tool of its kind in Japan that was evaluated via an RCT targeting older stroke patients. However, it is necessary to consider several limitations while interpreting the results. This study initially verified the genuine effects only of DA, so the intervention content consisted only of the distribution of DA or brochures, confirming their usage status. Therefore, although DAs are designed to promote SDM, offering them itself does not guarantee the implementation of SDM with families and various professionals. In the future, objective evaluation by family members and healthcare professionals is needed. Moreover, due to the limitations resulting from COVID-19, the intended sample size could not be achieved. This small sample size may have biased the study results and contributed to the lack of clear differences in effectiveness. Furthermore, when compared to intention-to-treat analysis, FAS analysis helps minimize the impact of dropouts and provides a more objective assessment of intervention effects. However, the possibility of underestimating the intervention effect must be considered due to the reduced number of subjects available for analysis. In addition, the dropout rate was high, approximately 40%, due to abrupt transfers because of COVID-19 and the facilities having large waiting lists. Thus, measures to reduce the dropout rate, such as implementing online assessments and optimizing the timing of the discharge questionnaire survey, should be considered. In this study, only one facility was used as the study site, and the severity of illness and ADL of the enrolled patients may not have been uniform. Facilities have many unique facility criteria and regional characteristics that may not be generalizable to other facilities. Thus, there is a need to increase research target facilities and study participants to generalize and standardize the findings and data and further understand the period and method of offering DA as well as the selection and content of target individuals.

## Conclusion

Our study showed that the use of DA with older stroke patients did not significantly decrease decisional conflict. In addition, while there was an increase in participation rate in decision-making, their active role did not significantly increase. However, DA showed a tendency to reduce uncertainty and promote participation rates, especially in participants living alone who were unable to decide their discharge destination. In the future, DA could be used with family members and professionals to promote the participation of older people in decision-making, thereby reducing conflicts in decision-making by carefully explaining the advantages and disadvantages of choices.

## Supporting information

S1 Checklist CONSORT checklist. (DOC)

S1 File A daily living function assessment. (PPTX)

S2 File Decision aid Japanese. (PDF)

S3 File Decision aid English. (PDF)

S4 File Manual for research assistants. (PDF)

S5 File Database. (XLSX)
