# Supplementary material for: A randomized controlled trial on the effects of decision aids for choosing discharge destinations of older stroke patients
Source: PLoS One. 2024 Jan 25;19(1):e0272115. doi: 10.1371/journal.pone.0272115 (PMC10810461; doi:10.1371/journal.pone.0272115)
Supplement: S1 File — (PPTX) [file pone.0272115.s002.pptx]

## Slide 1
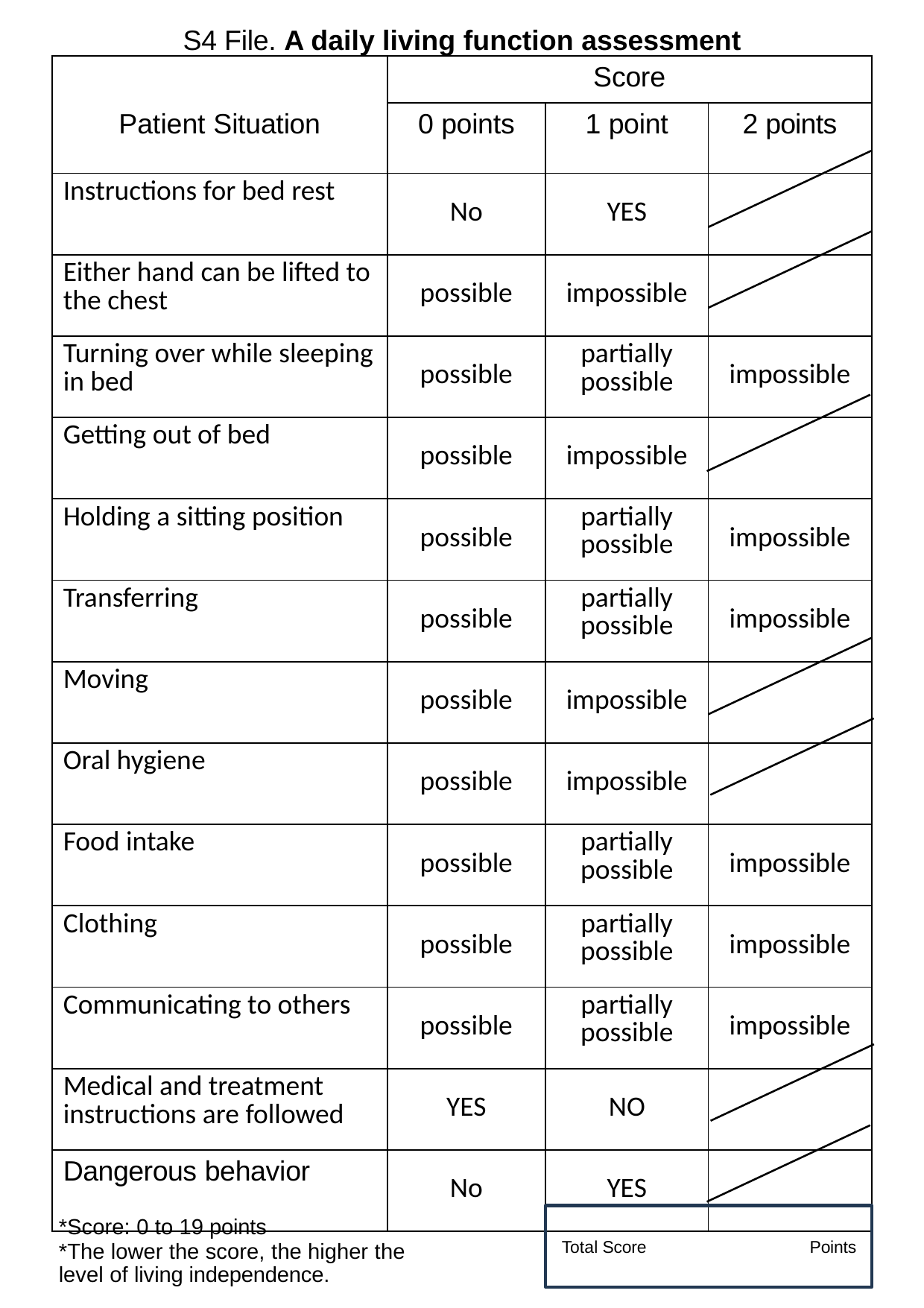

S4 File. A daily living function assessment
| Patient Situation | Score | | |
| --- | --- | --- | --- |
| | 0 points | 1 point | 2 points |
| Instructions for bed rest | No | YES | |
| Either hand can be lifted to the chest | possible | impossible | |
| Turning over while sleeping in bed | possible | partially possible | impossible |
| Getting out of bed | possible | impossible | |
| Holding a sitting position | possible | partially possible | impossible |
| Transferring | possible | partially possible | impossible |
| Moving | possible | impossible | |
| Oral hygiene | possible | impossible | |
| Food intake | possible | partially possible | impossible |
| Clothing | possible | partially possible | impossible |
| Communicating to others | possible | partially possible | impossible |
| Medical and treatment instructions are followed | YES | NO | |
| Dangerous behavior | No | YES | |
Total Score Points
*Score: 0 to 19 points
*The lower the score, the higher the level of living independence.
