# Supplementary material for: A randomized controlled trial on the effects of decision aids for choosing discharge destinations of older stroke patients
Source: PLoS One. 2024 Jan 25;19(1):e0272115. doi: 10.1371/journal.pone.0272115 (PMC10810461; doi:10.1371/journal.pone.0272115)

＜根拠に関連する引用・参考文献＞

1）西森旬恵：摂食・嚥下障害のある脳卒中患者の自宅退院に影響する要因, 愛媛県立医療技術大学紀要, 9(1), 11-16, 2012.

2）浅川育世他：回復期リハビリテーション病棟に入院した脳血管障害者の転帰に影響をおよぼす因子の検討ーFIM総得点90点以下の症例を対象にー, 理学療法科学, 23(4), 545-550, 2008.

3）津坂翠他：脳血管疾患等の患者が自宅退院するために必要な日常生活活動能力, 作業療法, 32(3), 256-261, 2013.

4）France Légaré, et al: Are you SURE? Assessing patient decisional conflict with a 4-item screening test, Can Fam Physician, 56, e308-14, 2010.

5）N. Brière, et al.: To get the care and services I need, should I stay in my home or move? , 2016.(Decision aid)

6）富山県ホームページ（健康、医療、福祉）：http://www.pref.toyama.jp/

memo

退院先を考えるガイドブック

脳卒中でご入院の方が退院先を考えるために

作成者：聖路加国際大学　看護学研究科　博士後期課程　青木 頼子

聖路加国際大学　看護情報学　教授　中山 和弘

作成日：2018年5月15日（情報が新しくなり次第随時更新の予定です）

平成27～30年　文部科学省科学研究費若手研究（B）No.15K20759による助成により作成されたものです。

この「退院先を考えるガイドブック」の開発は、医師、看護師、リハビリセラピスト（理学療法士、作業療法士、言語聴覚士）、社会福祉士の協力を得て作成しました。

退院先を考える ガイドブック

脳卒中でご入院の方が退院先を考えるために

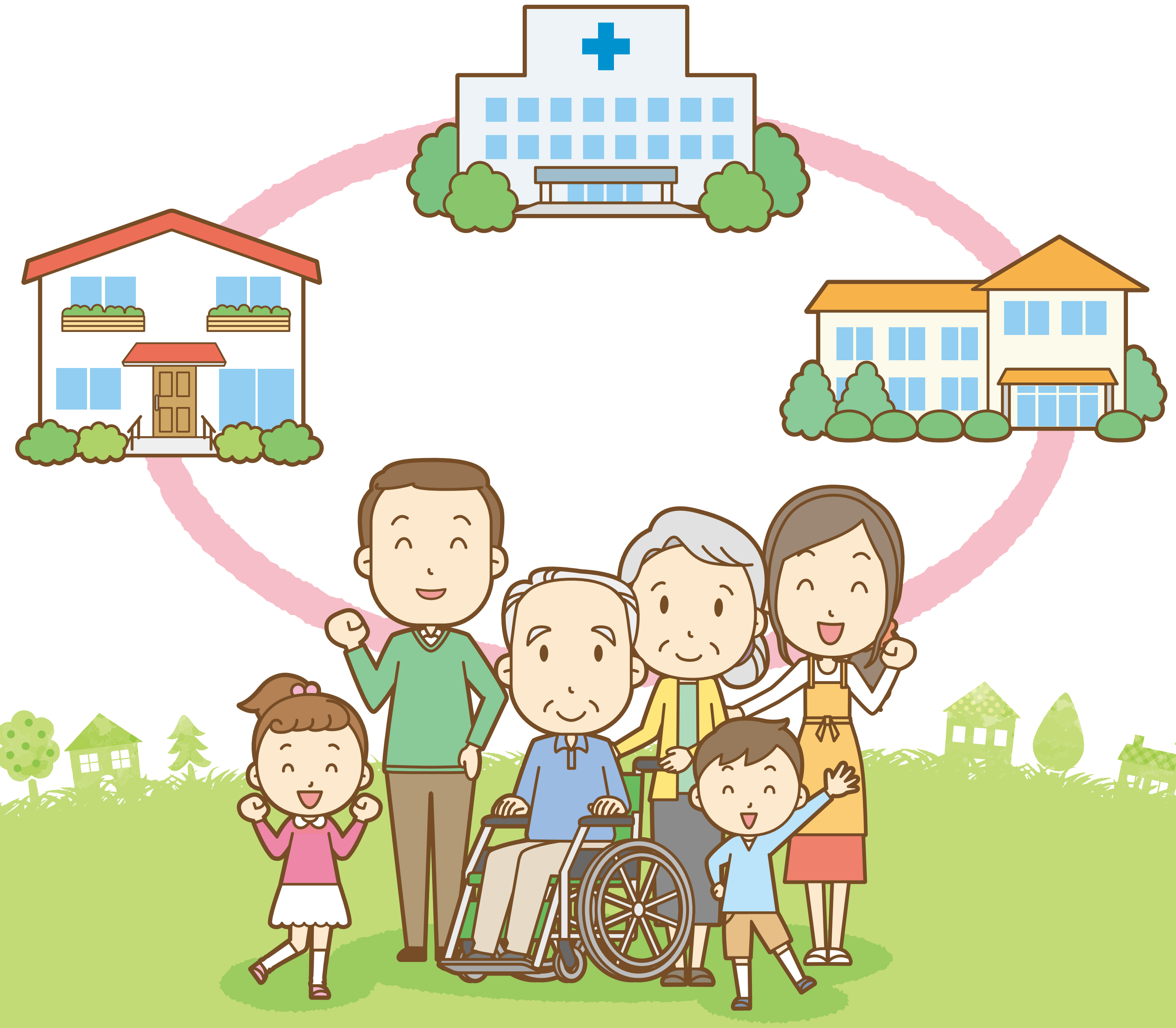

退院後のことが心配な方や退院先を迷っている方が、
ご家族や病院のスタッフと話し合うときにご利用ください。

このガイドブックは退院先として、
「入院前と同じ場所」か**「入院前と異なる場所」**かを
決める時に役立ちます。

どのような支援やサービスがあるのかを知り、
どの場所がより良い暮らしができるのかを
考えてみましょう。

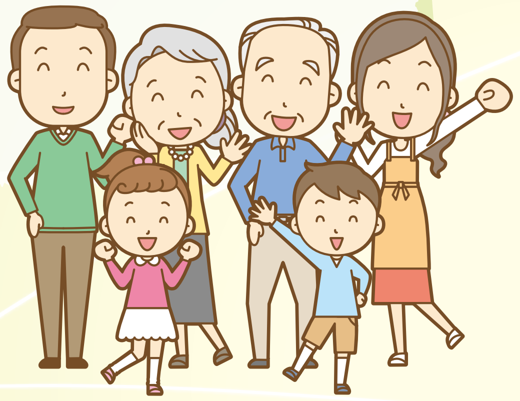

1から5の順番に読み進めることで、頭の整理ができます。

1 退院までの見通しを立てる P.3-4

2 サービスの種類や特徴を知る P.5-6

3 退院先の長所と短所を知る P.7-8

4 何が重要かを整理する P.9

5 準備ができたら退院先を決める P.10

◎あなたやご家族と一緒に退院先の準備を考えていく人を紹介します。

入院中

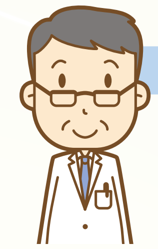

医 師
疾患の治療や説明をする。

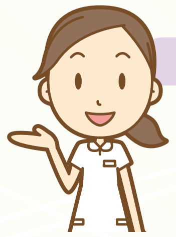

看護師
生活支援、看護介護指導、病気の管理や予防など。

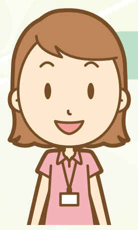

社会福祉士/MSW
退院への準備、経済的問題の解決など。

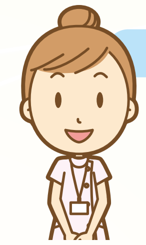

作業療法士/OT
日常生活ができるようにリハビリを行う。

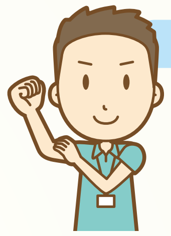

理学療法士/PT
基本動作ができるようにリハビリを行う。

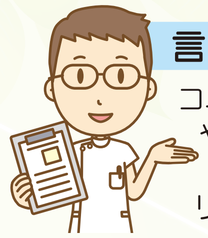

言語聴覚士/ST
コミュニケーションや食べることができるようにリハビリを行う。

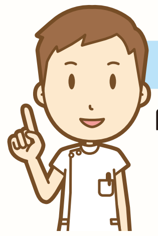

薬剤師
内服薬の内容、副作用、飲み方について説明をする。

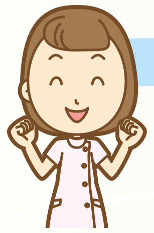

栄養士
食事の管理や栄養指導を行う。

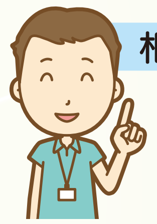

相談支援専門員
障害のある人の全般的な相談支援を行う。

退院後

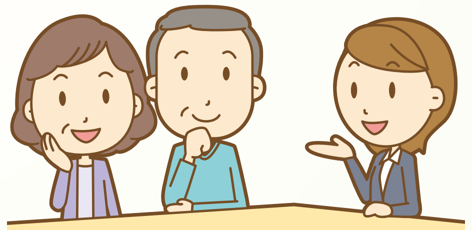

ケアマネジャー
要支援・要介護認定後に事業者との連絡調整をする。

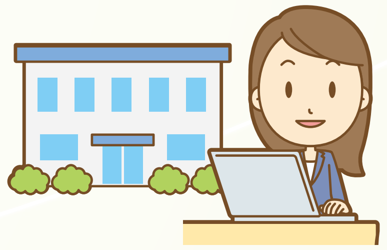

地域包括支援センター
介護・保健・福祉の相談やサービスを行う。

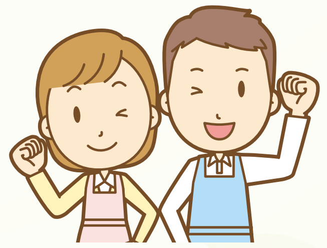

サービス事業者
訪問・通所・入所などのサービスを行う。

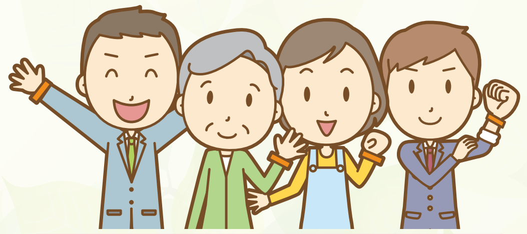

近所、民生委員、地域住民
ボランティア、NPO法人 など

1 退院までの見通しを立てる

今後、あなたがどのような生活を望むのかを考えながら、見通しを立ててみましょう。
（チェック ☑ してください）

■いま、希望する退院先はどちらですか？

□ **入院前と同じ場所**　　□ **入院前と異なる場所**

どこか ＿＿＿＿＿＿＿＿　　どこか ＿＿＿＿＿＿＿＿

■退院先を決めるとき、身近に支援してくれる方はいますか？

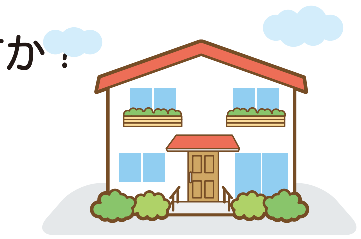

□ は　い　　□ いいえ

〈支援してくれる方について〉　その方にあなたの思いを知らせておくことも大切です。

誰ですか?（名前）	関係性（例：長女）	その方の考え	その方に自分の思いを伝えたか?	その方からの支援内容（例：買い物）
		入院前と □ 同じ場所 □ 異なる場所	□ 伝えた □ 伝えていない	
		入院前と □ 同じ場所 □ 異なる場所	□ 伝えた □ 伝えていない	

■だれと一緒に退院先を決めたいですか？

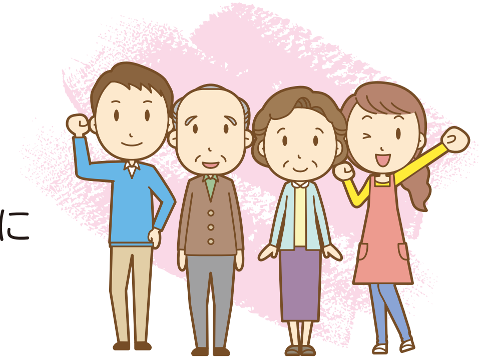

□ 自分一人　　□ 家族と一緒に

□ 医療者と一緒に　　□ 家族と医療者と一緒に

□（　　　　　　　　）にまかせたい

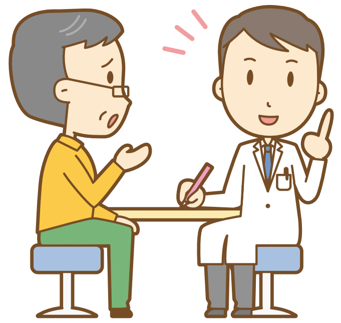

入院中には、つぎの手続きや準備を行います。
どのような手続きや準備が必要か、
いつ始めたらよいのかは人それぞれ異なりますので、
必要な時にスタッフからお伝えします。

介護保険の申請 変更申請

介護保険は介護が必要となったとき、1~2割の個人負担で利用できる制度です。訪問による聞き取りと主治医からの意見書で審査し、申請後30日以内に「自立」「要支援1･2」「要介護 1･2･3･4･5」の8段階で決定されます。

ケアマネジャーの決定 サービスの調整

支援が必要な場合、ご希望をお聞きしケアマネジャーを決定、退院先での生活がうまくいくようにサービスを考えていきます。

例）退院後に買い物をどうしようか困っている。
　　リハビリテーションを続けようか迷っている。など

住宅訪問と住宅の工事

退院先でより安全に暮らせるように、住宅に手すりを取り付けたり段差を解消したりします。必要であれば、病院のスタッフも住宅を訪問しお手伝いします。

外出・外泊訓練

退院先でよりスムーズに生活できるよう、必要な時期に外出や外泊の訓練をします。

介護技術の習得 内服・栄養指導 など

オムツ交換の仕方、食事介助の方法、吸痰の方法などの手技の練習を行います。

2 サービスの種類や特徴を知る

安心して退院先を選ぶためには、どのようなサービスが利用できるのかを知っておくことが大切です。介護度により、利用するかどうか、どれくらい利用できるのか異なりますので、詳しく知りたいときは病院の担当者にお尋ねください。

訪問サービスと通所サービス

✤主な介護保険サービス

生活のお手伝い

⇒訪問介護

ホームヘルパーが住宅に訪問し、入浴や食事、洗濯や買い物などを行います。
訪問入浴介護もあります。

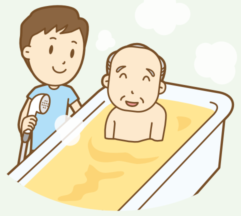

医療処置

⇒訪問診療、訪問看護

医師、看護師が住宅に訪問し、病状のチェック、吸痰や床ずれ、点滴などの医療処置を行います。

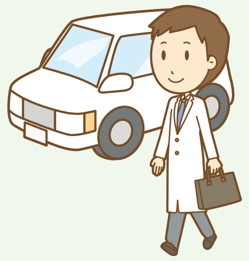

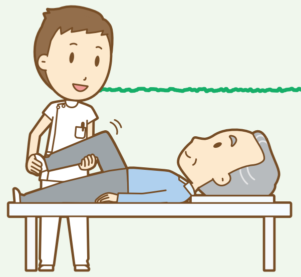

リハビリテーション

⇒通所・訪問リハビリテーション

自ら施設に出向いて、あるいはリハビリの専門家が住宅に訪問してリハビリを行います。

介護の一休み・宿泊

⇒ショートステイ

施設に短い間泊まって、食事、入浴、排泄などの支援や訓練を行います。

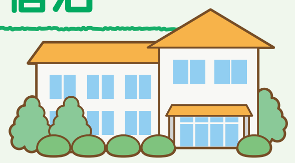

日中1人が心配 人と交流をしたい

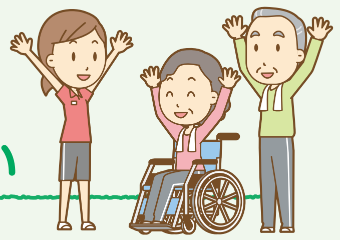

⇒デイサービス

施設で食事や入浴、レクリエーションなど、人との交流を日帰りで行います。

地域密着型のサービス

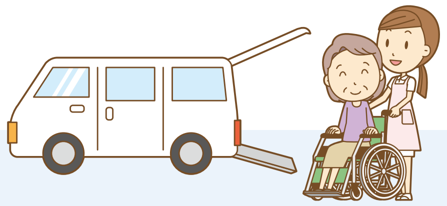

●原則としてお住いの市町村のみで利用できるサービスです

認知症対応型共同生活介護（グループホーム）

認知症の方が少人数で共同生活する住居です。

小規模多機能型居宅介護

通所を中心に、訪問、泊まりを組み合わせたサービスです。

※その他にも定期巡回や夜間対応型などのサービスもあります。

高齢者住宅・施設に入居するサービス

●入居後に介護度が進んだ場合、施設によって退去を求められることがあります。

●手すりの取り付けや段差なしなど、施設内は安全を考慮した環境です。

常に介護が必要

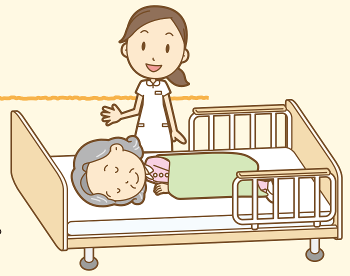

- 介護老人福祉施設（特養）

  要介護3以上の方が対象。

- サービス付き高齢者住宅
- 有料老人ホーム

  軽度～重度まで対応が可能ですが、全額自己負担。

  など

認知症への対応

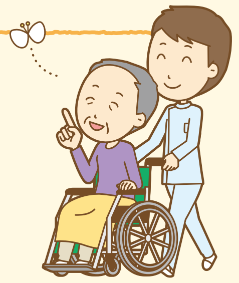

- 介護老人福祉施設（特養）
- 介護老人保健施設
- 介護療養型医療施設
- グループホーム
- サービス付き高齢者住宅
- 有料老人ホーム　など

リハビリテーション

- 介護老人保健施設　など

  状態が安定し、自分の家に帰ることを目的とした施設。

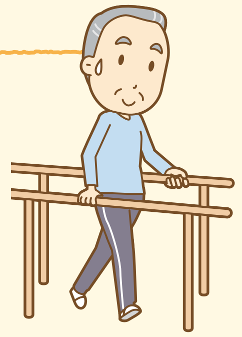

医療処置

- 介護療養型医療施設（医療者が24時間滞在）　など

  介護老人福祉施設と介護老人保健施設には、医療者が主に日中滞在。

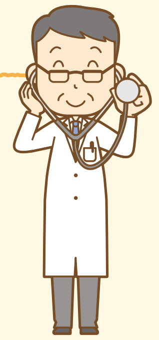

環境を整えるサービス

福祉用具を買う・借りる

福祉用具の貸し出し。排泄や入浴などに必要な用具の購入費を一部支給。

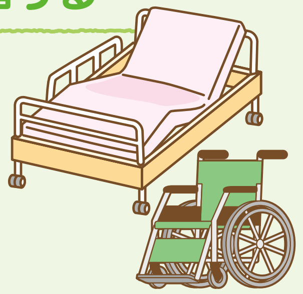

住宅の改修費

手すりを取り付けたり、段差をなくしたりする工事費用を一部支給。

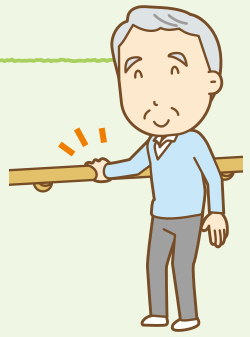

3 退院先の長所と短所を知る

〈**自分の家**〉とは、これまで自身が生活されてきた家のことです。
〈**病院・施設**〉とは、病院、介護老人福祉施設（特養）、介護老人保健施設、サービス付き高齢者住宅、有料老人ホームなどです。

	**入院前と同じ場所**		**入院前と異なる場所**		
	〈自分の家〉から 回復期病棟 から〈自分の家〉	〈病院・施設〉から 回復期病棟 から〈病院・施設〉	〈自分の家〉から 回復期病棟 から〈病院・施設〉	〈病院・施設〉から 回復期病棟 から〈自分の家〉	
生活程度	自立の状況に関わらず生活できます。<br>＊特にトイレ、移動、食べること、話すこと[1)2)3)]が重要と言われています。	病院・施設の担当者からの支援やサービスを受けながら生活できます。<br>＊退院後の身体の状態の変化によって、施設側から場所の移動を伝えられることもあります。	病院・施設の担当者からの支援やサービスを受けながら生活できます。	自立の状況に関わらず生活できます。<br>＊特にトイレ、移動、食べること、話すこと[1)2)3)]が重要と言われています。	生活程度
サービス・費用	訪問、通所、泊まりのサービスなどが利用できます。<br>**約10万円／1ヶ月**<br>（別に福祉サービス、おむつ代などの追加料金がかかります。）	サービス付き高齢者住宅や有料老人ホームでは、訪問、通所のサービスなどが利用できます。<br>病院や施設でのサービスは施設内で受けられます。<br>**特養　約13万円／1ヶ月**<br>**それ以外　約10～30万円／1ヶ月**<br>（居住費、食費、日常生活費込み）	サービス付き高齢者住宅や有料老人ホームでは、訪問、通所のサービスなどが利用できます。<br>病院や施設でのサービスは施設内で受けられます。<br>**特養　約13万円／1ヶ月**<br>**それ以外　約10～30万円／1ヶ月**<br>（居住費、食費、日常生活費込み）	訪問、通所、泊まりのサービスなどが利用できます。<br>**約10万円／1ヶ月**<br>（別に福祉サービス、おむつ代などの追加料金がかかります。）	サービス・費用
緊急時	訪問診療や訪問看護を依頼している場合には、医師や看護師が対応可能です。	病院・施設の担当者が対応可能です。	病院・施設の担当者が対応可能です。	訪問診療や訪問看護を依頼している場合には、医師や看護師が対応可能です。	緊急時
家族の支援	支援が必要な場合は、主に家族の支援が必要です。	家族に代わり、主に病院・施設の担当者が対応可能です。	家族に代わり、主に病院・施設の担当者が対応可能です。	病院・施設の担当者に代わり、主に家族の支援が必要です。	家族の支援
環　境	これまで通り、慣れ親しんだ場所と人に囲まれながら生活できます。	これまで通り、慣れ親しんだ場所と人に囲まれながら生活できます。<br>段差や手すりなど、安全面を考えた環境です。<br>集団生活となります。	新しい環境（場所、人など）に慣れる必要があります。<br>段差や手すりなど、安全面を考えた環境です。<br>集団生活となります。	慣れ親しんだ場所と人に囲まれながら生活することができます。	環　境
住宅の工事	身体の動きに合わせて、住宅の工事が必要です。	住宅の工事の必要はありません。	住宅の工事の必要はありません。	身体の動きに合わせて、住宅の工事が必要です。	住宅の工事

4 何が重要かを整理する

退院先を決めるうえで、ご自分が何を重要としているのかを整理しましょう。
それぞれの項目であなたが思う番号、1点(重要でない)〜5点(とても重要)に〇を付けてみましょう。

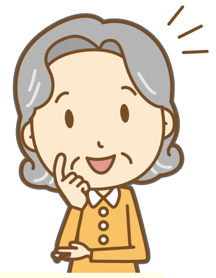

生活程度

Q1. あなたにとって、自分で生活できることはどれだけ重要ですか?

1	2	3	4	5
重要でない	←	・・・	→	とても重要

サービス・費用

Q2. あなたにとって、サービスの内容や費用はどれだけ重要ですか?

1	2	3	4	5
重要でない	←	・・・	→	とても重要

緊急時

Q3. あなたにとって、緊急時の対応はどれだけ重要ですか?

1	2	3	4	5
重要でない	←	・・・	→	とても重要

家族の支援

Q4. あなたにとって、家族の支援はどれだけ重要ですか?

1	2	3	4	5
重要でない	←	・・・	→	とても重要

環　境

Q5. あなたにとって、慣れ親しんだ場所と人はどれだけ重要ですか?

1	2	3	4	5
重要でない	←	・・・	→	とても重要

住宅の工事

Q6. あなたにとって、住宅の工事の有無はどれだけ重要ですか?

1	2	3	4	5
重要でない	←	・・・	→	とても重要

5 準備ができたら退院先を決める

あなたが決定した退院先についてまとめてみましょう。

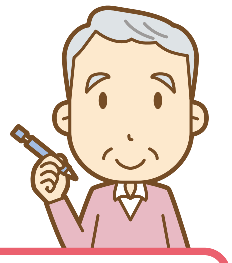

私は、このような生活がしたいから

どのような?＿＿＿＿＿＿＿＿＿＿

▼どちらかをチェック☑してください

☐ **入院前と同じ場所**　　☐ **入院前と異なる場所**

どこか＿＿＿＿＿＿　　どこか＿＿＿＿＿＿

に退院先を決定したいと思います。　日付：　年　月　日

最終的な決定が、後で変わっても大丈夫です。
心配なことや分からないことがあったら、いつでもスタッフにお尋ねください。

■退院先を決めるために、必要なことが準備[4)]できていたかどうかを確認してみましょう。（「はい」か「いいえ」で考えてみましょう）

1）この決定が自分にとって最良だと思いますか？

2）それぞれの退院先の特徴（長所と短所）について十分理解していますか？

3）退院後にどんな生活をしたいのか見通しがはっきりしていますか？

4）決定するうえで、十分な支援や助言はありましたか？

もし、上の4つの質問に1つでも「いいえ」があれば、
もう一度5〜6ページや7〜9ページに戻って考えてみましょう。

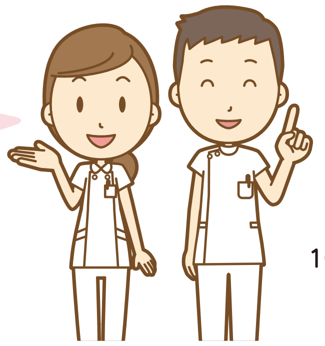

Supplement: S2 File — (PDF) [file pone.0272115.s003.pdf]
