# Supplementary material for: A randomized controlled trial on the effects of decision aids for choosing discharge destinations of older stroke patients
Source: PLoS One. 2024 Jan 25;19(1):e0272115. doi: 10.1371/journal.pone.0272115 (PMC10810461; doi:10.1371/journal.pone.0272115)

<Citations related to evidence - reference literature>

1) Nishimori, J.: Factors influencing home discharge of stroke patients with feeding and swallowing disorders, Bulletin of Ehime Prefectural University of Medical Technology, 9(1), 11-16,2012.

2) Ikuyo Asakawa et al: A study of factors influencing the outcome of patients with cerebrovascular disease admitted to a recovery-phase rehabilitation ward (FIM), Physiotherapy Science, 23(4), 545-550, 2008.

3) Tsusaka M. et al: Activities of daily living abilities required for patients with cerebrovascular disease and other conditions to be discharged home , Occupational Therapy,32(3), 256-261,2013.

4) France Legare, et al□ Are you SURE? Assessing patient decisional conflict with a 4-item screening test, Can Fam Physician, 56, e308-14, 2010.

5) N. Briere, et al.□ To get the care and services I need. Should I stay in my home or move? , 2016. (Decision aid)

6) Toyama Prefecture Home Page (Health, Medicine, Welfare)

http://www.pref.toyama.jp/

memo

Guidebook for thinking about
where to go after being discharged from the hospital

For people hospitalized with a stroke thinking about
where to go after being discharged from the hospital

Author: Yoriko Aoki, Doctoral Student, Graduate School of Nursing, St. Luke's International University
Kazuhiro Nakayama, Professor of Nursing Informatics, St. Luke's International University

Created on: May 15, 2018 (will be updated as soon as new information becomes available)

Supported by Grant-in-Aid for Young Scientists (B) No. 15K20759 from the Ministry of Education, Culture, Sports, Science and Technology, Japan, 2007-2008.

The guidebook was developed with the cooperation of physicians, nurses, physical therapists, occupational therapists, speech-language pathologists, and social workers.

Guidebook for thinking about where to go after being discharged from the hospital

For people hospitalized with a stroke thinking about where to go after being discharged from the hospital

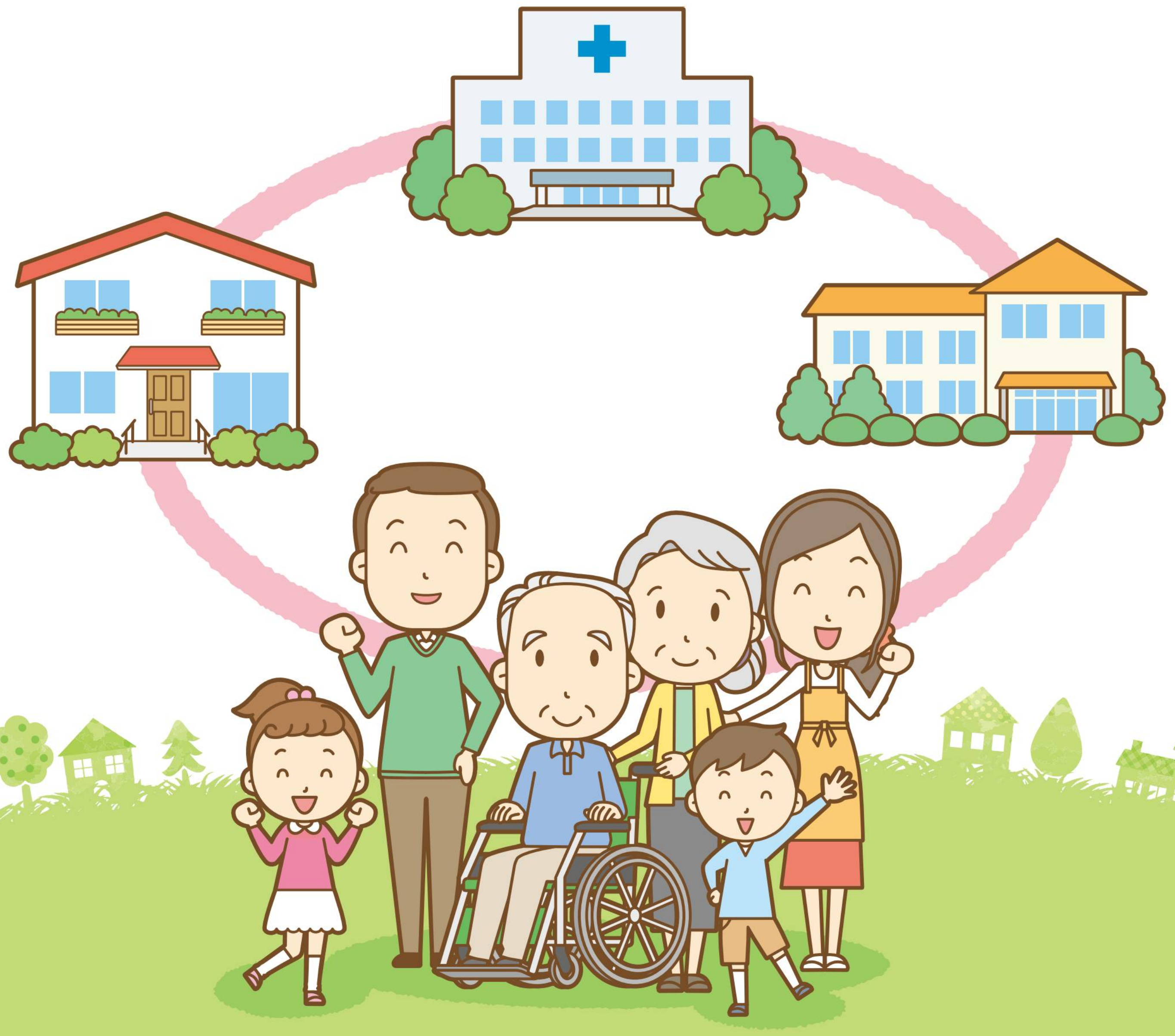

People who are worried about what to do after being discharged from the hospital or where to go should use this guidebook when discussing these issues with family members or hospital staff.

This guidebook will help you decide whether to leave the hospital in " same location as before your hospitalization " or " a different location from before your hospitalization ".

Find out what kind of support and services are available and consider which place would be a better place to live.

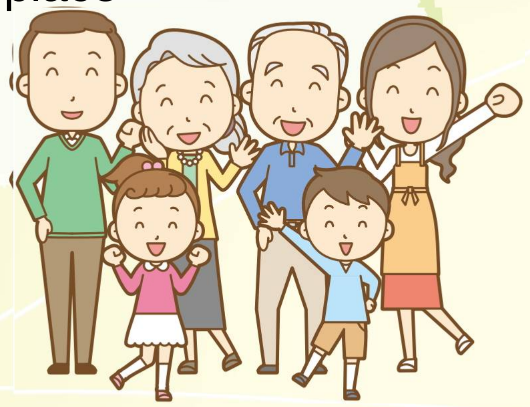

Reading them in order from 1 to 5 will help you get your head straight.

1	Planning your time until hospital discharge	P.3-4
2	Learning about the types and characteristics of service	P.5-6
3	Knowing the advantages and disadvantages of the hospital discharge destination	P.7-8
4	Organizing what is important	P.9
5	Decide your hospital discharge location once you are ready	P.10

◎Introduce someone who will work with you and your family to prepare for your discharge.

Hospitalized

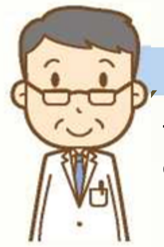

Physician

Treats and explains diseases

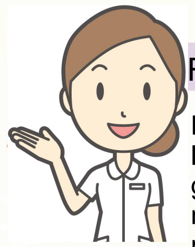

Registered nurse

Provide support for daily living, nursing care guidance, and disease management and prevention.

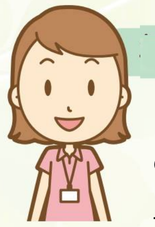

Social Worker/ MSW

Preparing for discharge from the hospital , resolving financial issues, etc.

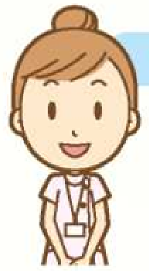

Occupational Therapist/OT

Rehabilitate patients to enable them to perform daily activities.

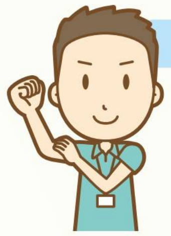

Physical Therapist/PT

Rehabilitate the patient to be able to perform basic movements.

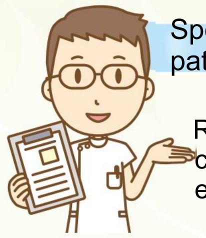

Speech - language pathologist/ST

Rehabilitation for communication and eating.

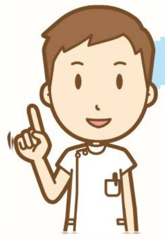

Pharmacist

Explain the contents, side effects, and dosage of oral medications.

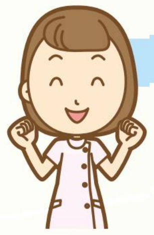

Nutritionist

Provides dietary management and nutritional guidance.

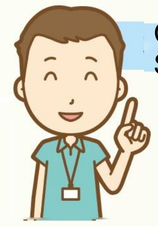

Consultant Support Specialist

Provide general counseling and support for people with disabilities.

After leaving hospital

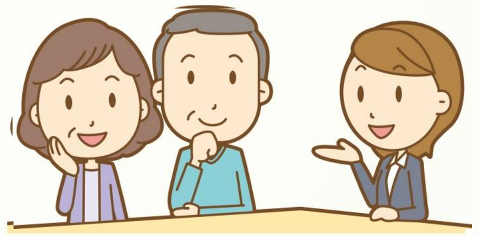

Care manager

Coordinate with service providers after the person is certified as needing support or care.

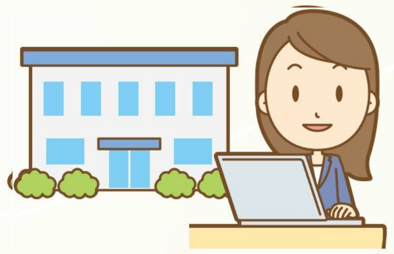

Community Comprehensive Support Center

Provide counseling and services in the areas of long -term care , health care, and social welfare.

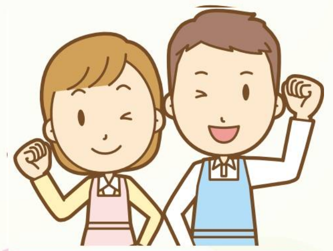

Service provider

Provides services such as home visits, day care, and residential care.

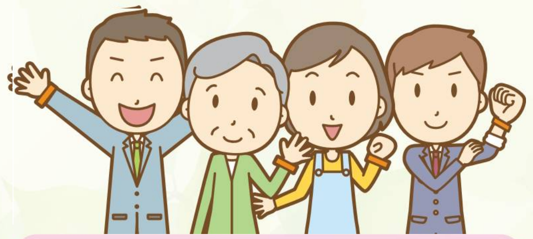

Neighbors, community members, local residents, volunteers, non-profit organizations, etc.

1 Planning your time until hospital discharge

Let's think about what kind of daily life you would like to have in the future and try to plan an outlook for the future.

Right now, where would you like your hospital discharge destination to be?

☐ Same location as before your hospitalization

Where? ______________________

☐ A different location from before your hospitalization

Where? ______________________

When planning where to go after your discharge, is there anyone close to you who can support you?

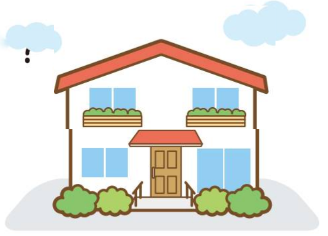

☐ Yes ☐ No

<About the supporting person>
It is also important to let this person know your thoughts.

Who is this person? (Name)	Relationship (e.g., eldest daughter)	That person's thoughts	Have you expressed your thoughts to that person?	Form of support from that person (e.g., shopping)
		Before hospitalization: ☐ Same location ☐ Different location	☐ Yes ☐ No	
		Before hospitalization: ☐ Same location ☐ Different location	☐ Yes ☐ No	

Who do you want to work with in deciding where your hospital discharge destination will be?

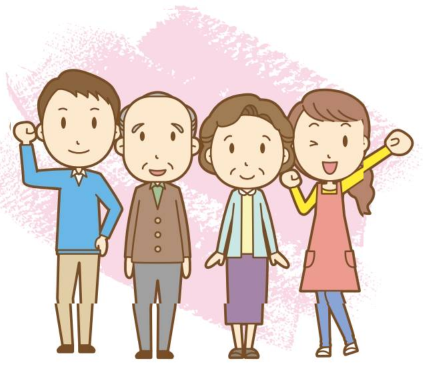

☐ Myself (alone) ☐ With family

☐ With a medical professional

☐ With a medical professional and family

☐ I want to entrust it to ( )

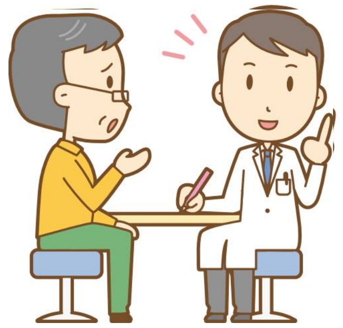

The following procedures and preparations will be performed during your hospitalization. The procedures and preparations required, along with when to start with these preparations, will vary by individual. As such, our staff will advise you as needed.

**Long-term care insurance application
Change application**

Long-term care insurance is a system that allows individuals to use the services by paying 10–20% out of pocket when long-term care is required. Examinations will be conducted via visit-based interviews and a written opinion from the attending physician; and a decision will then be made within 30 days of the application on an eight-level scale, ranging between: "independent", "in need of support 1-2", and "in need of long-term care 1-2-3-4-5."

**Care manager decision
Service adjustments**

If you need support, then we will listen to your wishes, select a care manager, and consider the services for helping you live a successful life at your hospital discharge destination.
Example: You are struggling with what to do about shopping; You are struggling with whether to continue rehabilitation; etc.

Home visits and home construction work

We will install handrails and remove steps in your home so that you can live more safely at your hospital discharge destination.

**Going out/
overnight stay training**

We will provide training for you if you want to go out and for overnight stays so that you can live more smoothly at your hospital discharge destination.

**Learning long-term care skils
Oral medication/nutrition guidance, etc.**

We will practice techniques such as how to change diapers, assist with meals, and how to suck sputum.

2 Learning about the types and characteristics of service

It is important to know what services are available so that you can choose a hospital discharge destination with peace of mind. Depending on your level of long-term care, whether you will use services and the extent to which you will use them will vary — so please ask hospital staff for details.

Visiting services and outpatient services

Main long-term care insurance services

Help with daily life

=> Visiting long-term care

The home helper visits the home and takes care of things like bathing, eating, doing laundry, and shopping. Visiting bathing care is also available.

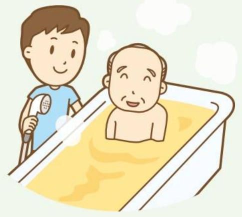

Medical treatment

=> Visiting medical treatment , visiting nursing care

The physician and nurse visit the home to check the patient's condition and provide medical treatment, such as sucking sputum, treating bedsores, and intravenous drip care.

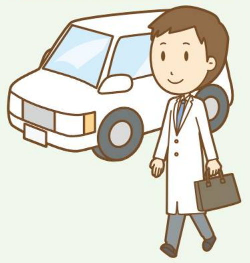

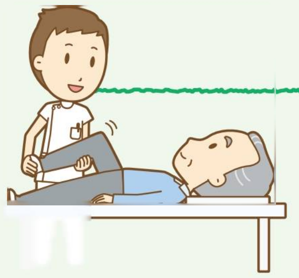

Rehabilitation

=> Outpatient/visiting rehabilitation

Rehabilitation can be conducted either by visiting the facility yourself, or by having a rehabilitation specialist visit your home.

Long-term care break/overnight stay

=> Short stay

You will stay at a facility for a short period of time and receive support or training with things such as eating, bathing, and using the bathroom..

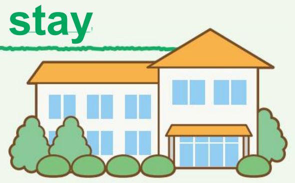

Concern about being alone during the day, A desire to interact with other people

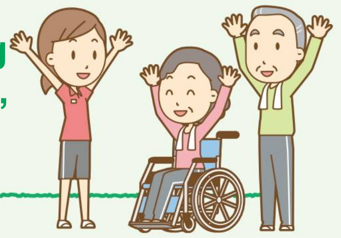

=> Day service

Interactions with other people at the facility — such as during meals, bathing, and recreation — are done as day trips.

Community-based services

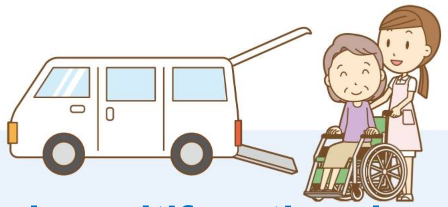

•As a general rule, this service is only available in the municipality where you live

Dementia-friendly communal living care (group home)

This is a residence where people with dementia live together in a small group.

Small-scale multifunctional home care

This is a service that combines visits and overnight stays, mainly through outpatient services.

*Other services — such as regular patrols and night-time services — are also available.

Services for moving into elderly housing/facilities

- **If your needed long-term care level increased after moving in, the facility may ask you to relocate.**
- The facilities are designed with safety in mind, such as the inclusion of handrails and a lack of steps.

Constant long-term care needed

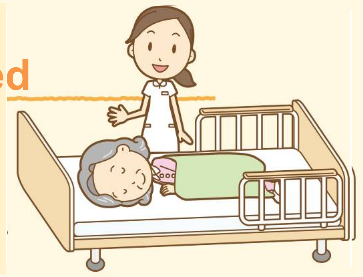

•Long-term elderly care welfare facility (special care facility)

For those who need long-term care (level 3 or higher).

- **Elderly housing with services**
- **Paid elderly home, etc.**

Mild to severe cases can be handled, but all expenses will come out of pocket, etc.

Dementia response

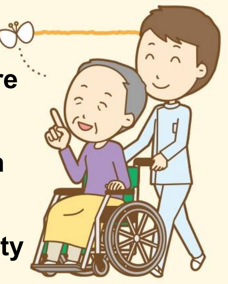

- **Long-term elderly care welfare facility (special care facility)**
- **Long-term elderly care health facility**
- **Long-term medical care facility**
- **Group home**
- **Elderly housing with services**
- **Paid elderly home, etc.**

Rehabilitation

•Long -term elderly health care facility, etc.

A facility for people to return to their own homes once their condition has stabilized.

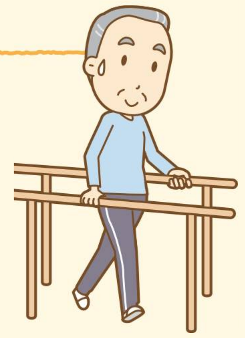

Medical treatment

•Long-term medical care facility (medical staff present 24 hours a day), etc.

Medical staff are mainly present during the day at long-term elderly care welfare facilities and long-term elderly care health facilities.

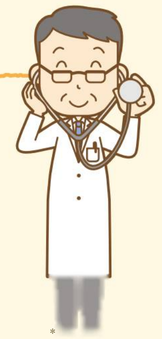

Services that improve the environment

Buying/renting welfare equipment.

Rentals of welfare equipment. Part of the cost of purchasing the equipment needed for using the bathroom , bathing , etc. will be provided.

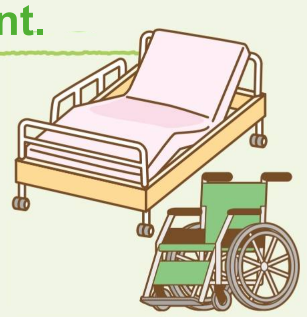

Medical treatment

Part of the construction costs for installing handrails and removing steps will be provided.

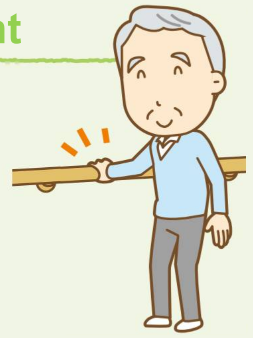

3 Knowing the advantages and disadvantages of the hospital discharge destination

<Own home> refers to the home in which you have lived until now.
<Hospital/facility> includes hospitals, long-term elderly care welfare facilities (special care facilities), long-term elderly care health facilities, elderly housing with services, and paid elderly homes.

	Same location as before hospitalization		Different location from before hospitalization	
	<Own home> to Convalescent ward to <Own home>	<Hospital/ facility> to Convalescent ward to <Hospital/ facility>	<Own home> to Convalescent ward to <Hospital/ facility>	<Hospital/ facility> to Convalescent ward to <Own home>
Level of daily life	You can live independently regardless of your circumstances.<br>*It is said that using the bathroom, moving around, eating, and speaking1) 2)3) are particularly important.	You can live your daily life while receiving support and services from the hospital and facility staff.<br>*Depending on changes in your physical condition after being discharged from the hospital, the facility may notify you of a change in location.	You can live your daily life while receiving support and services from the hospital and facility staff.	You can live independently regardless of your circumstances.<br>*It is said that using the bathroom, moving around, eating, and speaking1) 2)3) are particularly important.。
Services/ costs	Visiting, outpatient services, and overnight services are available.<br>Approximately 100,000 yen/month (additional fees concerning services such as welfare services and diaper fees will be charged.)	Visiting and outpatient services are available at elderly housing with services and paid elderly homes.Hospital and institutional services are available in the facility.<br>Special care facility: approximately 130,000 yen/month<br>Other: approximately 100,000–300, 000 yen/month (including housing, food, and daily living expenses)	Visiting and outpatient services are available at elderly housing with services and paid elderly homes.Hospital and institutional services are available in the facility.<br>Special care facility: approximately 130,000 yen/month<br>Other: approximately 100,000–300, 000 yen/month (including housing, food, and daily living expenses)	Visiting, outpatient services, and overnight services are available.<br>Approximately 100,000 yen/month (additional fees concerning services such as welfare services and diaper fees will be charged.)
Emergency	Physicians and nurses are available to respond if you request visiting medical care/nursing visits.	Hospital/facility staff are available to respond.	Hospital/facility staff are available to respond.	Physicians and nurses are available to respond if you request visiting medical care/nursing visits.
Family support	If you need support, then you will primarily need family support.	Hospital/facility staff will be available to respond on behalf of your family..	Hospital/facility staff will be available to respond on behalf of your family..	You will primarily need family support instead of hospital/facility staff.
Environment	You will be able to live your life as before, surrounded by familiar places and people.	You will be able to live your life as before, surrounded by familiar places and people.<br>The environment is designed with safety in mind, including considerations for steps and handrails. Group living is provided.	You will need to become accustomed to a new environment (location, people, etc.).<br>The environment is designed with safety in mind, including considerations for steps and handrails. Group living is provided.	You can live while surrounded by familiar places and people.
Housing construction	The housing will need to be constructed according to the mobility of the body.	There is no need for any housing construction.	There is no need for any housing construction.	The housing will need to be constructed according to the mobility of the body.

Level of daily life
Services/ costs
emergency
Family support
Environment
Housing construction

4 Organizing what is important

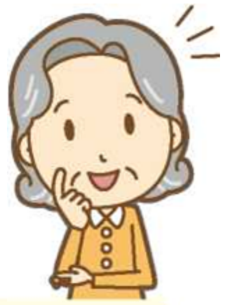

Let us organize what is important to you when deciding your hospital discharge destination .Please circle the number that you feel for each item — 1 (not important) to 5 (very important)

Level of daily life

Q1. How important is it to you to be able to support your own daily life?

1 2 3 4 5
Not important 1～Very important 5

Level of daily life

Q2. How important is the form of services or expenses to you?

1 2 3 4 5
Not important 1～Very important 5

Emergency

Q3. How important are emergency responses to you?

1 2 3 4 5
Not important 1～Very important 5

Family support

Q4. How important is family support to you?

1 2 3 4 5
Not important 1～Very important 5

Environment

Q5. How important are familiar places and people to you?

1 2 3 4 5
Not important 1～Very important 5

Housing construction

Q6. How important is the presence or absence of housing construction to you?

1 2 3 4 5
Not important 1～Very important 5

5 Decide your hospital discharge location once you are ready

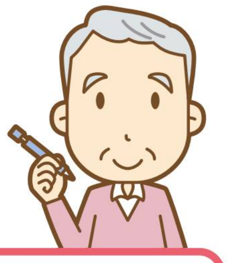

Let us summarize the hospital discharge destination that you have decided.

Because I want to live this kind of daily life

What kind? ______________________

▼Please check one of the following ☑

☐ Same location as before your hospitalization
Where? ______________________

☐ A different location from before your hospitalization
Where? ______________________

This is the hospital discharge destination that I would like to decide.

Date: (year) (month) (day)

You can change your final decision afterward.

Please feel free to ask staff members if you have any concerns or questions.

■ Let us check to see if you have prepared all the necessary items4) for deciding your hospital discharge destination (please think in terms of “yes” and “no”)

1) Do you think that this decision is best for you?

2) Do you fully understand the characteristics (advantages and disadvantages) of each hospital discharge destination?

3) Do you have a clear outlook on what kind of life you would like to live after being discharged from the hospital?

4) Did you receive sufficient support and advice in making your decision?

If you answered “no” to any of the four questions above, then please go back to pages 5–6 and 7–9 and think again.

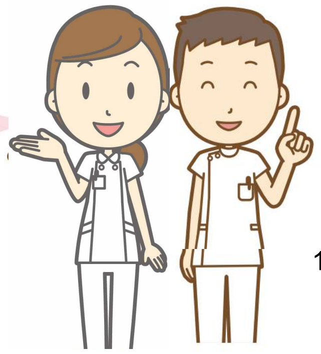

Supplement: S3 File — (PDF) [file pone.0272115.s004.pdf]
