# Supplementary material for: A randomized controlled trial on the effects of decision aids for choosing discharge destinations of older stroke patients
Source: PLoS One. 2024 Jan 25;19(1):e0272115. doi: 10.1371/journal.pone.0272115 (PMC10810461; doi:10.1371/journal.pone.0272115)
Supplement: S4 File — (PDF) [file pone.0272115.s005.pdf]

研究補助者用マニュアル（入院時、退院時共通）

1	**私にとってどのような選択肢(退院先)があるのかを知っている／分かった**<br><br>・選択肢とは、「入院前と同じ場所」と「入院前と異なる場所」の2つのこと。<br>・具体的な退院先は、自分の家、介護老人保健施設（老健）、介護老人福祉施設（特養）、サービス付き高齢者住宅、有料老人ホーム、介護療養型医療施設、認知症対応型共同生活介護（グループホーム）などがある。
2	**各選択肢(退院先の候補)の有益性(長所)を知っている／分かった**<br><br>・例えば、自分の家であれば、これまで通り、慣れ親しんだ人や場所に囲まれた生活を送ることができ、自立の状況に関わらず、選択可能な場所であり、生活費は施設を利用するよりも安価となる。<br>・サービス付き高齢者住宅や有料老人ホーム、老健や特養などの施設では、施設の担当者からの支援やサービスを受けながら生活することができ、緊急時の対応や住宅の工事の心配がいらず、家族の支援も少なくてすむ。
3	**各選択肢(退院先の候補)の危険性(短所)を知っている／分かった**<br><br>・例えば、自分の家であれば、生活が自分である程度できる状態であることが望ましいことや、家族の支援が必要となること、身体の動きに合わせて住宅の工事が必要となる。<br>・施設では、自分の家よりも1ヶ月の生活費がかかること、新しい人や場所に慣れる必要がある。
4	**どの有益性(長所)が自分にとって最も重要であるのかはっきりしている／はっきりした**<br><br>・自分の家の場合、<br>「生活程度」では、自分でできる範囲により、入院前に近い生活を送ることができる。自立の状況に関わらず、選択可能な場所である。<br>「サービス・費用」では、訪問、通所、泊りのサービスを受けることができ、施設を利用するよりも安価である。<br>「緊急時」では、自身で対応となるが、訪問診療や訪問看護を依頼している場合には、医師や看護師が対応可能である。<br>「家族の支援」では、慣れた人からの援助を受けることができる。<br>「環境」では、慣れ親しんだ場所と人に囲まれながら生活することができる。<br>「住宅の工事」では、身体の動きや安全面に不都合がなければ、工事は最小限に抑えることができる。<br><br>・施設の場合、<br>「生活の程度」では、介護や認知症が重度になっても施設の担当者からの支援やサービスを受けて生活することができる。<br>「サービス・費用」では、サービスが施設内で受けることができる。<br>「緊急時」では、病院や施設の担当者によって対応可能である。<br>「家族の支援」では、家族に代わり主に施設の担当者が支援するため、家族の負担を軽減できる。<br>「環境」では、段差の少ないバリアフリーの環境で安全に過ごすことができる。<br>「住宅の工事」では、住宅の工事の必要はない。
5	**どの危険性(短所)が自分にとって最も重要であるのかはっきりしている／はっきりした**<br><br>・自分の家の場合<br>「生活程度」では、介護度や認知度が重度になった場合には、サービスや家族などの支援が増える。<br>「サービス・費用」では、訪問型のサービスでは、自分の家にサービス業者が訪れることになり、通所型では、送り迎えの時間などに制約を伴うことがある。費用は、サービスを利用する分金額が高くなる。<br>「緊急時」では、主にご自身や家族がおられる場合には家族がまず対処する必要がある。<br>「家族の支援」では、支援が必要な分、家族の支援が必要となる。<br>「環境」では、住んでいる地域によって、サービスが利用しづらくなる場合もある。身体の動きによって、今まで慣れて生活してきた環境でも転倒しやすかったり、ぶつかったり危険を生じることがある。<br>「住宅の工事」では、身体の動きに合わせて、手すりや段差解消などの工事が必要となる。<br><br>・施設の場合<br>「生活の程度」では、主に利用されている方々との集団生活となるため、施設でのスケジュールに合わせて生活することになる。<br>「サービス・費用」では、自分の家での生活にかかる費用と比較して、金額は高くなる。<br>「緊急時」では、施設の担当者から連絡が入ることになる。<br>「家族の支援」では、施設が家族から遠方になると通うことに困難を生じる。<br>「環境」では、施設への引っ越しをし、新しい環境や人に慣れる必要がある。<br>「住宅の工事」では、工事の必要はなく、段差のないバリアフリーの環境であるが、一般的な高さで設置されているため、逸脱した場合には、補助具が必要となる場合もある。

6	**有益性（長所）、危険性（短所）のどちらがより重要であるかはっきりしている／はっきりした**<br><br>・前記の4と5の内容を比較し、どちらがより重要かを査定する。<br>何が最も重要だと考えているのかが答えられたら、はっきりした（明確になった）と判断する。<br>例）麻痺により、トイレ行動に手すりが必要なため、住宅の工事は必要だと感じている。家族への介護の負担はかけたくないとは思っているが、サービスの費用が高くなると支払いに困る　など。
7	**選択をするための十分な支援を他者から受けている／受けた**<br><br>・退院先を考える時に、支援を他者から受けたかどうかであり、他者とは、家族、親戚、友人、病院内の他の患者や家族、病院の担当者などを指す。
8	**他者からの圧力を受けることなく選択している／選択できた**<br><br>・他者からの圧力とは、例えば家族（子供）が自分への相談なしに施設入所を決めてきた、子供、親戚、他患者、病院の担当者、ケアマネジャーなどから介護が大変になる、家での生活は無理だと言われた　など
9	**選択をするための十分な助言を得ている／得た**<br><br>・退院先を考える時に、家族や病院の担当者、病院内の他の患者や家族、友人などから助言を受けたかどうか。
10	**どの退院先の候補が自分にとって最良であるかはっきりしている／はっきりした**<br><br>・退院先の候補は、「入院前と同じ場所」か「入院前と異なる場所」の2つのことである。<br>具体的に自分の家、サービス付き高齢者住宅、有料老人ホーム、老健、特養など場所を特定していても良い。<br>自分にとって最良とは、自身の身体症状や現状を受け止めている場合と受け止めていない場合があるが、自身の主観で自分にとって一番良いと考えている退院先の候補が明確かどうかで判断する。
11	**何を選択すべきか／選択すべきだったかについて自信がある**<br><br>・選択とは、退院先の「入院前と同じ場所」か「入院前と異なる場所」のことであり、具体的な場所を答えても良い。10番の設問と同様に、自身の身体症状や現状を受け止めている場合と受け止めていない場合があるが、10番は主観的な明確さを尋ねており、11番は自信について尋ねている。
12	**退院先を決定をするのは、私にとって容易である／容易であった**<br><br>・主観的に退院先を決定することが容易であったかどうかを尋ねている。
13	**十分な情報を得て選択できる／選択できたと感じている**<br><br>・家族、親戚、友人、他患者や家族、スタッフなどから自身が十分と考える情報量を得た上で、退院先を決定できると感じているかどうかを尋ねている。
14	**私の退院先は自分にとって何が重要かを考えて決定している／決定できた**<br><br>・設問の4、5、6番の長所と短所を比較した上で、自分にとって一番何を重要としながら決定できたのかという価値観を基盤とした意思決定ができたかどうかを問う設問である。
15	**私の退院先の決定は変わることはないと思う**<br><br>・退院先を決めた後の揺らぎや納得の度合いを尋ねる設問である。
16	**今の自分の退院先決定に満足している**<br><br>・今現在の退院先の意思決定の満足の度合いを尋ねる設問である。
